# Deep soil exploration vs. topsoil exploitation: distinctive rooting strategies between wheat landraces and wild relatives

**DOI:** 10.1007/s11104-020-04794-9

**Published:** 2020-12-21

**Authors:** Alireza Nakhforoosh, Kerstin A. Nagel, Fabio Fiorani, Gernot Bodner

**Affiliations:** 1 Division of Agronomy, Department of Crop Sciences, University of Natural Resources and Life Sciences, Vienna (BOKU), Konrad Lorenz-Straße 24, A-3430 Tulln an der Donau, Austria; 2 Global Institute of Food Security, University of Saskatchewan, Saskatoon, SK S7N 0W9 Canada; 3 IBG-2: Plant Sciences, Forschungszentrum Jülich GmbH, 52425 Jülich, Germany

**Keywords:** Root phenotyping, Root system ideotypes, Deep rooting, Wheat wild relatives, Durum landraces, Drought tolerance, Breeding

## Abstract

**Aims:**

Diversity of root systems among genetic resources can contribute to optimize water and nutrient uptake. Topsoil exploitation vs. deep soil exploration represent two contrasting ideotypes in relation to resource use. Our study reveals how rooting patterns changed between wheat wild progenitors and landraces in regard to these ideotypes.

**Methods:**

Root (partitioning, morphology, distribution, elongation, anatomy) and shoot traits (dry-matter, leaf area, assimilation) of durum landraces, wild emmer and wild einkorn from Iran, Syria, Turkey and Lebanon were phenotyped using the GrowScreen-Rhizo platform. Distinctive rooting patterns were identified via principal component analysis and relations with collection site characteristics analyzed.

**Results:**

Shoot trait differentiation was strongly driven by seed weight, leading to superior early vigor of landraces. Wild progenitors formed superficial root systems with a higher contribution of lateral and early-emerging nodal axes to total root length. Durum landraces had a root system dominated by seminal axes allocated evenly over depth. Xylem anatomy was the trait most affected by the environmental influence of the collection site.

**Conclusions:**

The durum landrace root system approximated a deep soil exploration ideotype which would optimize subsoil water uptake, while *monococcum*-type wild einkorn was most similar to a topsoil exploiting strategy with potential competitive advantages for subsistence in natural vegetation.

**Supplementary Information:**

The online version contains supplementary material available at 10.1007/s11104-020-04794-9.

## Introduction

Sustainable intensification has been defined as novel strategic framework for post-green revolution agriculture (Collette et al. 2011). It aims to provide answers to two key challenges: reversion of the decreasing trend in yield advance for major staple crops observed since the 1990s (Brisson et al. 2010) and reduction of yield dependence on high external inputs (fertilizers, pesticides, irrigation). Measures to achieve these objectives rely on an optimization of natural potentials of crops and cropping systems in order to effectively transform available soil resources into high and stable yields.

In the context of breeding, sustainable intensification combines modern technologies with plant biodiversity to detect and make use of underutilized genetic resources for introgression of novel traits. It has been demonstrated that broadening the genepool can contribute to advanced cultivars with improved stress resistance (Hoisington et al. 1999; Xie and Nevo 2008). For trait-based breeding, that aims to systematically improve the physiological potential of crops, Reynolds and Langridge (2016) defined six major steps: (a) crop ideotype design, (b) exploration of genetic resources that might bear the desired traits, (c) phenotyping of the collected accessions for the targeted traits, (d) genetic analysis, (e) hybridization and selection, and (f) multi-location testing.

With water being globally the main limiting resource for food production (Hanjra and Qureshi 2010), the high uptake efficiency of stored soil water resources by annual food crops is fundamental to ensure high yields in drought-prone cropping environments (Blum 2009). Thus, it would be of particular interest to identify plant genetic resources characterized by traits that convey superior resistance against tissue dehydration, thereby delay stomata closure, enhance assimilation and ultimately improve yield (Mujeeb-Kazi et al. 2009).

Following the physiological breeding framework of Reynolds and Langridge (2016), a deep root system can be defined as one essential component of a drought resistant crop ideotype because it controls the access to and uptake of available water resources from soil (Kell 2011; Bodner et al. 2015). Root system ideotypes and the single traits composing them have been elaborated based on model simulations. For example, a “deep, steep and cheap” ideotype has been postulated for environments limited in water/nitrate (mobile resources; Lynch 2013). Stored soil water is of particular importance for plants growth under limited in-season rainfall. At such sites, stress avoidance and yield levels largely depend on the efficiency of plants to efficiently explore the soil profile for available water resources while minimizing the competition between shoot and root for assimilates (e.g. Lilley and Kirkegaard 2011; Tron et al. 2015). This is achieved by a “deep, steep and cheap” root system that combines increased root length density, a high angle/gravitropic response of seminal roots, few and long lateral roots, long root hairs with high longevity and cortical aerenchyma (Meister et al. 2014; Lynch 2019).

Globally 43% of the land area is estimated to be P limited, particularly highly weathered tropical and subtropical (Du et al. 2020). Phosphorous is an immobile plant nutrient where topsoil root foraging has been identified as crucial acquisition strategy for plants (Lynch and Brown 2001). Topsoil foraging root systems result from shallow branching angles of primary (tap+basal) root axes with strong lateral branching and high denisty/length of root hairs.

Overall, root system ideotypes provide a theoretical synthesis of current plant physiological understanding and thereby facilitate targeted trait selection via phenotyping platforms.

There are indications that root systems of modern cultivars insufficiently meet the ideotypic requirements for efficiently exploring/exploiting soil resources. For example, a study of Waines and Ehdaie (2007) demonstrated the decreasing trend of root biomass in post-green revolution wheat genotypes, assuming that this may result in sub-optimum root system size for water and nutrient uptake. Recently this trend was also confirmed for a set of Swiss winter wheat genotypes covering 100 years of breeding history (Friedli et al. 2019). However, for modern cultivars it is crucial that superior soil resource acquisition by roots is not mediated by higher belowground assimilate allocation and thus competing sink strength between shoot/grain and root (Palta and Turner 2019).

Plant genetic resources with high and still largely unexploited root diversity might therefore contribute to develop novel cultivars with root systems resembling the postulated ideotypes. Root system characterization of a spring-wheat sub-set of the cultivated wheat collection demonstrated high diversity in root traits with rooting depth significantly affected by the region of origin among the investigated accessions (Narayanan et al. 2014). Krishnamurthy et al. (2003) found differences in root growth dynamics between chickpea cultivars and wild relatives (time to linear root elongation phase and rooting depth). Higher allocation of root axes in topsoil layers was identified as trait of interest for nutrient foraging from wild accessions in a soybean core collection (Zhao et al. 2004). In a screen of Mexican wheat landraces Reynolds et al. (2006) described some accessions with particularly high ability to explore deep soil moisture resources. For durum wheat, Nakhforoosh et al. (2014) observed that modern cultivars had a higher specific root length compared with wild relatives, leading to an effective utilization of available soil moisture, while the latter were distinguished by higher biomass allocation to roots. In addition to morphological changes in root architecture during wheat domestication also functional changes have occurred, e.g. root hydraulic conductivity increased from wild to modern cultivated species (Zhao et al. 2005). Gioia et al. (2015) reported higher root functionality for nitrogen uptake of modern durum wheat compared to wild and domesticated emmer, which was attributed to the breeding process under high nitrogen supply.

Overall, exotic parents (e.g. *Aegilops* ssp.) and landraces have already been successfully used in wheat breeding programs for abiotic stress resistance (e.g. water, salt; Colmer et al. 2006; Placido et al. 2013; Reynolds et al. 2006). A main controversy on the importance of root diversity for breeding is related to phenotyping challenges, leading Reynolds and Tuberosa (2008) to consider direct selection for variation in root characteristics as impractical. However, substantial advance in root imaging during the last decade has (partially) overcome this phenotyping bottleneck. Currently a wide range of root phenotyping platforms is available, ranging from high-throughput seedling root methods to advanced 3D structural-functional approaches (Zhu et al. 2011). Weak correlation between seedling root screens on artificial media with field-grown root systems has led to the establishment of field-near platforms based on soil-filled containers (termed rhizotrons or rhizoboxes) where plants can be grown over longer time periods (e.g. Bodner et al. 2017; Nagel et al. 2012). This is particularly relevant for monocots with predominantly post-embryonic shoot-borne roots that cannot be directly inferred from seminal characteristics of seedling plants and thus require phenotyping until the tillering stage and later (Watt et al. 2013).

Aiming to identify the specific role of domestication and region of origin in durum wheat collection, Nakhforoosh et al. (2016) – based on automated shoot phenotyping - identified four distinct water use strategies. They concluded that breeding advance towards modern cultivars has improved water use efficiency and vigorous growth by high assimilation rates per unit leaf area, while the overall transpiring (and assimilating) leaf surface was reduced, i.e. change in structure was compensated by higher functionality. Based on these results, we hypothesize that transition from a highly branched shoot architecture in wild wheat species towards a reduced number of dominant main stems in cereal evolution/breeding (Doust 2007) is reflected in a concomitant modification of the root system.

In this study, we therefore aim to identify changes in ideotypic wheat rooting patterns at the transition of wild (diploid and tetraploid) progenitors to durum wheat landraces. In addition, our accession should demonstrate how geographic origin combines with the different genetic background of species collected at the same sites of origin. We expect that a better understanding of how ploidy (diploid vs. tetraploid), domestication (wild vs. cultivated) and geographic origin have shaped root systems could facilitate the identification of promising genetic resources that meet the “deep, steep and cheap” paradigm of water efficient root systems.

## Material and methods

### Plant materials and regions of origin

Wheat genotypes from four different eco-hydrological regions were selected from the U.S. National Plant Germplasm System (GRIN). Based on geographical coordinates of the collection sites, one wild diploid einkorn (depending on availability at collection sites: wild einkorn_red_, *Triticum urartu,* 2n = 2x = 14, AA for Syria and Iran; wild einkorn *Triticum monococcum subsp. aegilopoides* 2n = 2x = 14, A^m^A^m^ for Lebanon and Turkey; Michikawa et al. 2019), one wild tetraploid emmer (*Triticum turgidum subsp. dicoccoides*; 2n = 4x = 28; AABB) and one domesticated tetraploid durum landrace (*Triticum turgidum subsp. durum*; 2n = 4x = 28; AABB; landrace definition, see Zeven 1998) were collected at the same region of origin (Table 1); i.e. in total 12 genotypes.

**Table 1:** Investigated durum wheat landraces and wild relatives and characteristics of sites of origin

Accession number (name, ploidy)	Country	Seed weight (g 100 seeds^−1^)	Latitude (°N)	Longitude(°E)	Altitude (m)	Aridity index	Soil type	T_min_ (°C)	T_max_(°C)	Rain(mm d^−1^)	PET(mm d^−1^)
*Triticum urartu (Wild einkorn*_*red*_*,* AA*)*											
PI 428316 (G3220)	Iran	1.2	34.1	46.5	1650	0.34	Calcisol	7.0	17.2	0.9	2.9
PI 487269 (SY 20123)^*^	Syria	1.9	32.6	36.8	1450	0.20	Vertisols	11.4	22.1	1.5	4.0
*Triticum monococcum (Wild einkorn,* A^m^A^m^*)*											
PI 427996 (G3120)	Lebanon	1.3	33.5	35.9	1141	0.66	Leptosol	7.4	20.6	2.5	3.5
PI 427538 (G1892)	Turkey	1.3	37.2	39.7	720	0.40	Calcisol	5.5	22.1	1.4	3.8
*Triticum turgidum subsp. dicoccoides (Wild emmer, AABB)*											
PI 428016 (G1392)	Iran	1.7	34.4	46.1	1458	0.18	Leptosol	12.7	27.2	1.2	4.1
PI 487251 (SY 20013)^*^	Syria	2.4	32.5	36.6	920	0.27	Leptosol	7.0	17.1	0.9	2.9
PI 428133 (G3100)	Lebanon	2.7	33.5	35.9	1141	0.66	Leptosol	7.4	20.6	2.5	3.5
PI 554582 (84TK110–087)^*^	Turkey	2.1	37.8	39.8	775	0.37	Leptosol	9.8	23.0	1.3	3.8
*Triticum turgidum subsp. durum (Durum landrace, AABB)*											
PI 621590 (IWA8608790)^*^	Iran	6.7	34.1	46.5	1336	0.34	Calcisol	5.5	22.1	1.4	3.8
PI 182699 (B-1)	Syria	4.6	32.8	36.5	821	0.27	Cambisol	8.7	20.2	1.3	3.1
PI 182667 (9923)	Lebanon	7.5	33.8	35.9	866	0.51	Vertisols	7.8	21.2	1.7	3.4
PI 166321 (Beyaz)	Turkey	4.9	37.2	39.8	568	0.29	Calcisol	11.4	22.1	1.5	4.0

*Long accession names shortened as IWA790, 13_ SY, 84TK, 23_SY; countries’ names abbreviated as Iran (IR), Syria (SY), Lebanon (LB) and Turkey (TK); wheat species abbreviated as MON (*monococcum*), URT (*urartu*), DIC (*dicoccoides*), DRM (*durum*). PET is daily mean potential evapotranspiration

According to the UNEP-aridity index (Middleton and Thomas 1992) the accessions originated from semi-arid to sub-humid climates with predominant soil types being lime accumulation soils typical for dry climates (Calcisol), shallow soils of mountainous areas (Leptosol), soils of temperate highland climates (Cambisol) and expansive-clay soils (Vertisol; Harmonized World Soil Database; Nachtergaele et al. 2010). Meteorological site characteristics during the vegetation period have been obtained from the local climate estimator NewLocClim (Grieser et al. 2006).

### Phenotyping platform and growth conditions

For phenotyping the accessions, 20 medium-sized seeds of each genotype were selected (average seed weight cf. Table 1) and grow on moist filter paper in Petri dishes for 6 days at 4 °C in the dark. Upon emergence, uniformly germinated seeds were transplanted (at 2 cm depth) into rhizotrons (90 cm depth × 60 cm width × 3.4 cm inner space; soil volume: 18.360 cm^3^) filled with dark peat soil (Graberde; Plantaflor Humus, Vechta Germany; containing ~120 mg L^−1^ N, ~8.7 mg L^−1^ P, ~141.1 mg L^−1^ K; sieved <8 mm) as substrate and drainage holes at the bottom. The rhizotrons were placed into the GrowScreen-Rhizo root phenotyping platform at 43° inclination angle as described by Nagel et al. (2012).

The experimental setup was a randomized complete block design with six replicates and one plant per rhizotron. Plants were grown under optimum moisture (water content at field capacity) for four weeks (24 September to 22 October 2015) in the PhyTec Experimental Greenhouse at Plant Sciences (IBG-2) Institute, Forschungszentrum Jülich. All rhizotrons were watered twice a day with 200 ml of tap water to keep the moisture content at field capacity (mean potential evapotranspiration [Penman-Monteith] during the experiment was 6.9 ± 1.5 mm d^−1^). The environmental growth conditions in the greenhouse were 22.9 ± 0.7 °C daily mean temperature, 55.5 ± 6.1% average air humidity, 36.7 ± 8.2 MJ m^−2^ d^−1^ global radiation (with supplementary illumination sources) and a light duration of 14 h (06:00 to 20:00 local time). The experiment was terminated 28 days after sowing (DAS) when the root system of the first genotype approached the bottom of the rhizotron.

### Measurement of shoot traits

Plant phenological growth stage was recorded five times in the course of the experiment using the BBCH coding system (Lancashire et al. 1991).

Shoot growth was estimated via leaf area expansion. Leaf area (LA) was obtained from non-destructive measurements of leaf length (L) and width (W) at 8 and 15 DAS and following the relation of LA = 0.858 x L x W (Gioia et al. 2015). At the time of harvest (28 DAS) leaf area was measured destructively using a leaf area meter (LI-3100 Area Meter). Leaf area duration (LAD) was calculated by integrating measured leaf area over time.

Gas exchange was measured at 20–23 DAS (between 09.00 and 13.00 h) to determine stomata conductance, assimilation and transpiration rates (here only assimilation rate, A_max_, is reported). Measurements were performed on the last fully developed leaf of each plant using a LICOR LI-6400 infrared gas analyzer (leaf chamber: 1 × 3 cm). Settings of the leaf chamber were: light intensity of 1000 μmol m^−2^ s^−1^ PAR (greenhouse ambient light: 216 ± 51 μmol m^−2^ s^−1^), chamber temperature of 20 °C (resulting in a steady leaf temperature of 23 ± 0.5 °C), humidity of 60 ± 5% and CO_2_ of 400 μmol mol^−1^. Adjustment of plants to reach steady values of assimilation rate at the indicated chamber settings was about 10 min. In case of leaf width less than the chamber size (< 1 cm width), measurements were corrected with the effective leaf area inside the chamber.

### Root phenotyping and root anatomical traits

Visible roots at the rhizotron front plate were imaged by the GrowScreen-Rhizo platform two times a week, giving a total number of eight images per rhizobox. Roots were analyzed using the image analysis software GROWSCREEN-Root to extract time series of root length and convex hull area of: (i) the embryonic roots (primary radicle and basal root axes; subsequently termed seminal roots), (ii) the lateral roots emerging from the embryonic seminal root axes (subsequently termed lateral roots), and (iii) the post-embryonic shoot-borne nodal root axes (subsequently termed nodal roots; all definitions following Hochholdinger and Tuberosa 2009; Zobel and Waisel 2010 and Orman-Ligeza et al. 2013). The phenotyping platform and image analysis procedure are described in detail by Nagel et al. (2012).

After termination of the experiment at 28 DAS, plant roots were carefully washed and stored in alcohol solution (30% mixture of ethanol and distilled water) in a refrigerator at 4 °C for subsequent root morphological analysis by WinRhizo (Regent Instruments Inc., Québec City, Canada; scanner resolution; 400 dpi). Here only total root length of the whole root system were included in further analyses. To determine root biomass, root samples were dried at 60 °C and weighted after 48 h.

Xylem vessel diameter of deep reaching seminal roots is an essential functional trait for subsoil water usage (Richards and Passioura 1989; Richards 2006). Anatomical root samples were obtained from three seminal axes sampled at 6 cm distance from the base of the main axis. The root specimens were transversely cross-sectioned in 20 μm thick segments by a microtome and subsequently stained by phloroglucinol-HCl to contrast the xylem cell walls (red-violet) against the background for counting the numbers of meta- and protoxylem vessels (Lux et al. 2015). Digital images of the cuts were taken on a ‘Zeiss Axiophot 2’ fluorescent microscope at ×100 magnification equipped with a ‘Zeiss Axiocam’ 208 color digital camera and images were processed with ImageJ (Rueden et al. 2017) to measure the area of xylem vessels.

### Statistical analysis

Analysis of variance (ANOVA) was performed for each trait to test genotype, species and origin effects using the MIXED procedure in SAS 9.2 (SAS institute, Inc., Cary, NC).

The model for analyzing the randomized complete block design (RCBD) is given by

$$ {y}_{ij}=\mu +{\alpha}_i+{r}_j+{e}_{ij} $$

where y_ij_ is the respective plant trait for the ith genotype in the jth block, μ is the general mean, α_i_ is the main effect of genotype (fixed), r_j_ is the main effect of block (fixed; Piepho et al. 2003) and e_ij_ is the random error term. Due to unbalanced data with regard to genotypes (einkorn species) per site, (i) restricted maximum likelihood (REML) to estimate variance components, (ii) the Kenward-Roger method was selected for degree of freedom approximation, and (iii) mean comparison was done with adjusted means (LSMEANS) using Tukey’s test (Piepho et al. 2003; Spilke et al. 2005). Specific hypotheses on trait distinction due to species and region of origin were tested by linear contrasts using the CONTRAST statement in the MIXED procedure. Regression analysis by PROC REG was used to assess relations among traits.

For analysis of root depth distribution and root growth, functional parameters were obtained. Depth distribution was described by an asymptotic function for cumulative root length proportion over depth captured by the *β*-parameter (Jackson et al. 1996). Root growth of seminal axes followed an exponential function rising to maximum, while growth of lateral and nodal root axes was described by a sigmoid function. Curve fitting was done using the NLIN procedure of SAS and subsequently parameters were compared statistically via analysis of variance (PROC GLM).

Similarity among genotypes based on a multivariate analysis of their root traits was assessed by principle component analysis (PCA) and biplots (Bodner et al. 2013). Correlations between shoot and root traits characterizing the accessions and meteorological parameters from their collection sites were tested using PROC CORR.

## Results

### Shoot growth and development

Results from measurements of shoot traits are given in Table 2. Genotypic differences were mostly driven by the effect of species, except for assimilation rate where also the region origin was identified as a significant factor.

**Table 2:** Average values of shoot traits of durum wheat landraces and wild relatives from different sites of origin

Genotype	Origin	Shoot Traits^1^			
		SH_DM_(g)	LAD(cm^2^d)	A_max_(μmol m^−2^ s^−1^)	BBCH(−)
Wild einkorn^†^					
G3220 *(uratu)*	IR	0.047	152.7	55.4	20.5
23_SY *(uratu)*	SY	0.132	343.5	20.0	22.0
G3120 *(mono.)*	LB	0.095	272.4	11.9	21.3
G1892 *(mono.)*	TK	0.100	270.4	31.3	21.8
Wild emmer					
G1392	IR	0.155	325.6	30.9	22.2
13_SY	SY	0.145	427.4	9.2	21.3
G3100	LB	0.133	386.5	7.2	22.0
84TK	TK	0.120	347.4	24.4	21.8
Durum landraces					
IWA790	IR	0.438	1108.0	10.5	22.3
B-1	SY	0.200	651.6	1.7	21.5
9923	LB	0.242	671.2	8.4	21.8
Beyaz	TK	0.268	627.9	13.0	22.2
s.e.d^2^		0.0314	69.51	7.13	0.677
Genotype ^3^		<.0001	<.0001	< 0.001	0.370
Species		<.0001	<.0001	<0.001	0.258
Origin		0.071	0.062	<.0001	0.862

^1^SH_DM_, Shoot dry matter; LAD, Leaf area duration; A_max_, Maximum assimilation rate; BBCH, phonological development stage at final harvest

^2^s.e.d., Tukey H.S.D. adjusted standard error of differences for genotype effect

^3^*p* value for Genotype, Species (*T. turgidum* subsp*. durum,* subsp*. dicoccoides, T. monococcum, T. urartu*) and Origin (Iran, Lebanon, Syria and Turkey)

†To designate the difference between *T. urartu* and *T. monococcum* subsp*. aegilopoides,* we use the common name Wild einkorn_red_ and Wild einkorn respectively

Aboveground dry-matter varied in the range of 0.047 g (Iranian wild einkorn_red_ G3220 to 0.438 g plant^−1^, Iranian durum landrace IWA790). The genetic background was the key driver for shoot dry-matter accumulation explaining 57.6% of total variance. Linear contrasts revealed that particularly the domesticated durum wheat landraces differed from the wild progenitors (0.29 vs 0.10 g plant^−1^). Origin had only a minor and non-significant influence on shoot dry-matter.

Similarly to shoot dry-matter, also leaf area duration was driven mostly by genetic background (65.0% of total variance). Also here the durum landraces contrasted to wild progenitors in leaf area duration (LAD; 764.7 vs. 315.7 cm^2^ d). Within the wild types, wild emmer had significantly higher LAD (369.4 cm^2^ d) compared to *urartu*-type wild einkorn_red_ (187.0 cm^2^ d), while the *monococcum*-type was in-between. Again, region of origin was an insignificant contributor to variability in LAD.

Light-saturated CO_2_-assimilation rate A_max_ differed among the accessions with both species significantly contributing to the overall variance (species explained 27.5% and origin 24.7% of variance; interaction between species and origin was non-significant, *p* = 0.0781). Linear contrast analysis identified A_max_ of the *urartu*-type wild einkorn_red_ as significantly higher (37.9 μmol m^−2^ s^−1^) than all others, with *monococcum*-type wild einkorn (21.4 μmol m^−2^ s^−1^) and wild emmer (18.0 μmol m^−2^ s^−1^) and at an intermediate position and durum landraces with the lowest mean A_max_ values (8.0 μmol m^−2^ s^−1^). Concerning the effect of origin, Iranian and Turkish accessions (32.1 and 28.6 μmol m^−2^ s^−1^) had significantly higher A_max_-values than Syrian and Lebanese accessions (10.3 and 14.7 μmol m^−2^ s^−1^).

Differences in phenology (BBCH) were not significant during the comparatively short duration of the phenotyping experiment. All shoot and root parameters were thus registered at the same development stages.

**Fig. 1:**
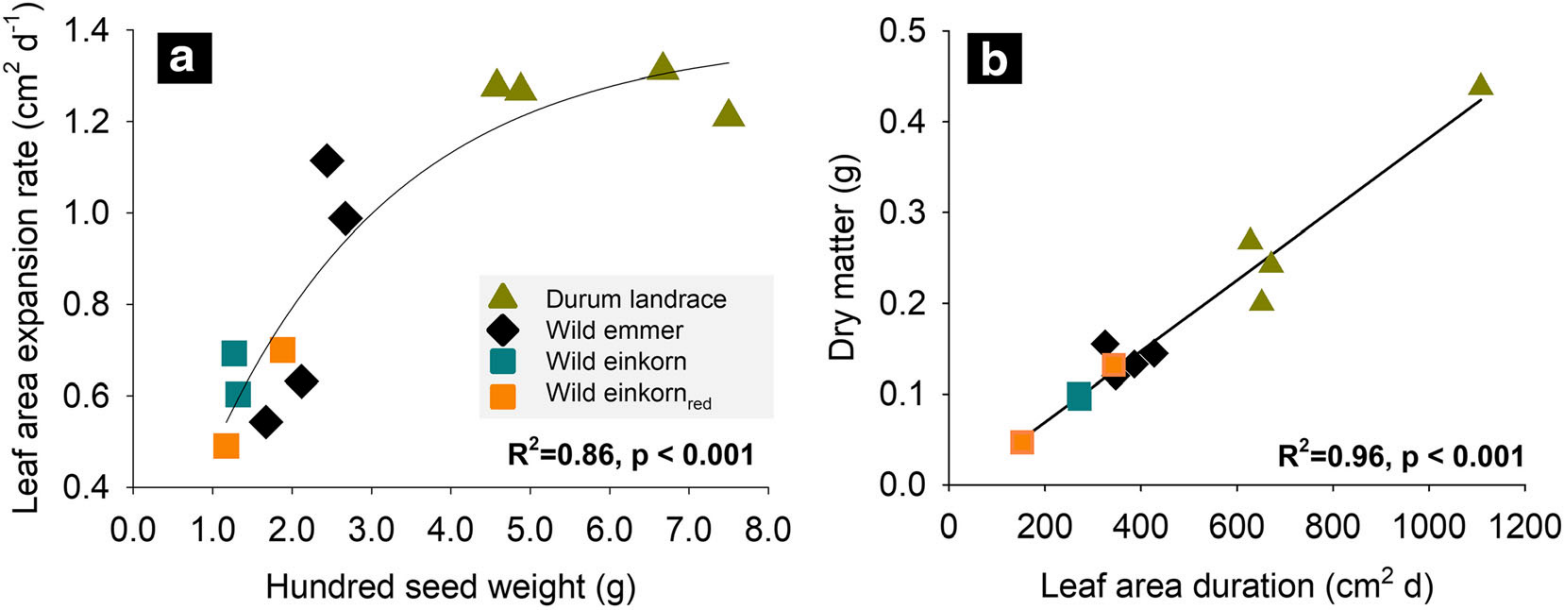
Traits driving growth during early stage phenotyping of wheat genetic resources (mean values of genotypes). **a** Influence of seed size (100 seed weight) on initial leaf area expansion rate; **b** Leaf area duration effect on shoot dry-matter accumulation

Figure 1 shows results from regression analysis to identify the main drivers of shoot dry matter accumulation until developmental stage BBCH 22. Genotypes with higher hundred seed weight had a significant higher initial leaf area expansion (leaf area per day; similar for measurements at 8 and 15 DAS) with the effect following an asymptotic pattern (Fig. 1a). The functional form suggests that this is most evident for the slowly expanding leaves of wild progenitors with small sized seeds, while disappearing for the larger sized seeds of durum landraces. With a larger assimilating leaf area, also the accumulated early stage shoot dry matter was higher with all durum landraces at the upper end of the collection (Fig. 1b). Although varying in A_max_, we could not determine a significant relation of dry matter with assimilation rate from our data (R^2^ = 0.14, *p* = 0.3260).

### Root system traits

#### Root visibility and whole root system length

**Fig. 2:**
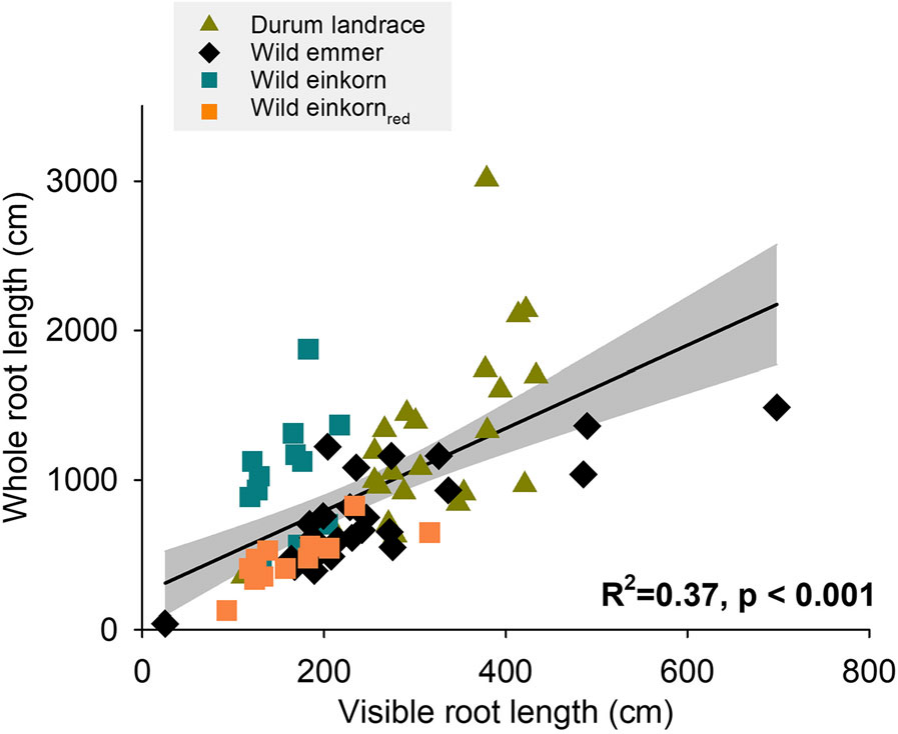
Relation between visible root length and total root length of the whole root system

Phenotyping growth and architecture of visible root axes of rhizotron-grown plants by the GrowScreen-Rhizo phenotyping platform captured between 16% (Turkish wild einkorn G1892) and 45% (Syrian wild emmer SY20123) of total root length. The relation between visible root length at the single rhizotron observation windows and post-experimentally measured (washed) total root length was significant (Fig. 2) with an R^2^ of 0.38. The destructively determined whole root length was mostly related to the rhizotron surface-scanned seminal length, which constituted in average 71.2% of visible length. We notice that some genotypes differed significantly in the percentage of visible root length, particularly the Turkish and Lebanese wild einkorn with lowest and the Syrian and Turkish wild emmer with highest visibility.

All root system traits measured at the end of the experiment (whole root system, visible traits, anatomy) are given in Table 3.

**Table 3:** Root traits of durum wheat landraces and wild relatives from different sites of origin

Genotype	Origin	Whole root traits^1^			Visible root traits^2^				Anatomy^3^			
		RL(cm)	R/S(−)	SRL(cm g^−1^)	RL_sem_(cm)	RL_lat_(cm)	RL_nod_(cm)	Conv. A. (cm^2^)	MX_A_ PX_A_ (mm^2^ *×* 10^−3^)		MX_N_ PX_N_ *(N)*	
Wild einkorn												
G3220 *(uratu)*	IR	394.7	0.26	375.0	85.9	25.5	21.2	236.5	2.83	0.90	3.0	7.5
23_SY *(uratu)*	SY	552.2	0.15	280.3	152.9	19.4	29.7	607.0	2.57	1.61	1.7	8.5
G3120 *(mono.)*	LB	1078.3	0.21	555.2	86.0	50.2	33.3	403.1	2.08	0.92	1.0	8.5
G1892 *(mono.)*	TK	1011.8	0.20	536.8	97.0	28.7	22.9	501.1	2.19	1.06	1.5	8.5
Wild emmer												
G1392	IR	933.8	0.15	398.0	189.7	5.7	27.5	759.3	3.29	1.93	2.0	9.3
13_SY	SY	771.9	0.20	258.5	163.1	157.1	30.9	698.8	2.21	1.28	1.2	7.5
G3100	LB	856.8	0.19	343.5	117.9	153.7	22.1	567.8	2.47	1.80	1.2	8.4
84TK	TK	522.1	0.17	290.9	137.5	19.0	22.3	472.3	4.01	1.63	2.8	8.3
Durum landraces												
IWA790	IR	1874.4	0.10	423.0	299.5	23.3	35.3	1751.6	2.80	1.28	1.8	8.3
B-1	SY	895.3	0.12	365.4	237.6	30.9	29.0	745.1	2.79	1.52	1.8	8.8
9923	LB	966.4	0.13	313.8	290.7	6.0	27.3	735.0	3.14	1.47	1.3	8.7
Beyaz	TK	1180.4	0.11	386.4	238.2	5.1	22.6	844.2	3.14	1.69	2.0	9.2
s.e.d^4^		222.7	0.023	25.4	25.8	32.6	10.4	281.7	0.45	0.12	0.43	0.37
Genotype ^5^		<.0001	<.0001	<.0001	<.0001	<.0001	0.938	<.0001	0.165	<.0001	0.032	0.023
Species		<.0001	<.0010	<.0001	<.0001	0.0003	0.856	<.0001	0.136	<.0001	0.985	0.149
Origin		0.0875	0.221	<.0001	0.084	0.004	0.661	0.002	0.069	<.0001	0.024	0.027

^1^RL, Root length; R/S, root-to-shoot ratio; SRL, Specific root length

^2^RL_sem_, Seminal root length; RL_lat_, Lateral root length (lateral roots emerging from seminal axes); RL_nod_, Nodal root length (definition of axes types cf. Material and Methods); Conv. A., convex hull area

^3^MX_A_, Metaxylem vessel area; PX_A_, Protoxylem vessel area; MX_N_, Metaxylem vessel number; PX_N_, Protoxylem vessel number. Measured at three seminal root axes sampled at 6 cm distance from the base of the main axis

^4^s.e.d.,Standard error of differences for genotype effect

^5^*p* value for Genotype, Species (*T. turgidum* subsp*. durum,* subsp*. dicoccoides, T. moncoccum, T. urartu*) and Origin (Iran, Lebanon, Syria and Turkey)

The length of the entire root system varied significantly among genotypes. The Iranian durum wheat landrace IWA790 had the highest total root length, while the Iranian *urartu*-type wild einkorn_red_ G3220 was at the lower end. Linear contrasts showed that at species level durum landraces had similar total root length with *monococcum*-type wild einkorn accessions, followed by wild emmer and the *urartu*-type wild einkorn_red_ at the lower end. Origin had no significant influence on total root length.

#### Root dry matter allocation

Partitioning of dry matter between root and shoot (root-shoot ratio, R/S) and within the root system (specific root length, SRL) are reported in Table 3. Both traits (R/S, SRL) differed significantly between genotypes. The proportion of total dry matter allocated to the root systems decreased from wild progenitors (average: 0.19, with non-significant differentiation between wild species) to durum landraces (0.12). Highest R/S was found for the Iranian wild einkorn_red_ accession and lowest for the Iranian durum landrace. Region of origin of the genotypes did not significantly affect dry matter partitioning between root and shoot.

For SRL, both genotype and region of origin had a significant influence. The *monococcum*-type wild einkorn accessions were significantly higher in their SRL (546.0 m g^−1^) compared to all others, with durum landraces in between the wild emmer and *urartu*-type wild einkorn_red_. Specifically, the Syrian wild emmer and wild einkorn_red_ had low SRL, resulting in an overall significantly lower SRL (301.4 m g^−1^) of species with Syrian origin compared the other collection sites.

We notice that root length distribution over diameter classes followed a bimodal pattern with a main peak at a root diameter of 0.4 mm (between 21.2 and 25.3% of total root length) and secondary peak at 0.8 mm (cf. Supplementary Fig. S1).

#### Length and proportion of different root classes

At the level of root classes (visible seminal, lateral and nodal root axes), durum landraces had an average higher seminal root length compared to the wild relatives (266.5 cm vs. 128.7 cm). Among the wild relatives, seminal root length was higher for wild emmer compared to the wild einkorn accessions (152.0 cm vs. 105.5 cm), although the Syrian *urartu* was an exception with a comparatively high seminal root length. Visible seminal axes length did not indicate significant influences by the region of origin.

The length of lateral roots, emerging from the seminal axes, showed a marked difference of the Lebanese and Syrian wild emmer genotypes compared to the other accessions (155.4 cm vs. 21.4 cm). Comparison at the ploidy level via linear contrasts indicated that on average the lateral root length of wild emmer was highest (83.9 cm), *monococcum*-type wild einkorn in between (39.5 cm) and *durum* landraces (22.5 cm) and *urartu*–type wild einkorn_red_ (16.3 cm) at the lower end. For lateral root length, also a significant contrast among accessions due to their region of origin was found (Lebanon and Syria higher than Turkey and Iran).

Nodal roots constituted the smallest proportion of visible axes at the early phenological stage of the experiment and did not show any significant differentiation in length among the accessions.

**Fig. 3:**
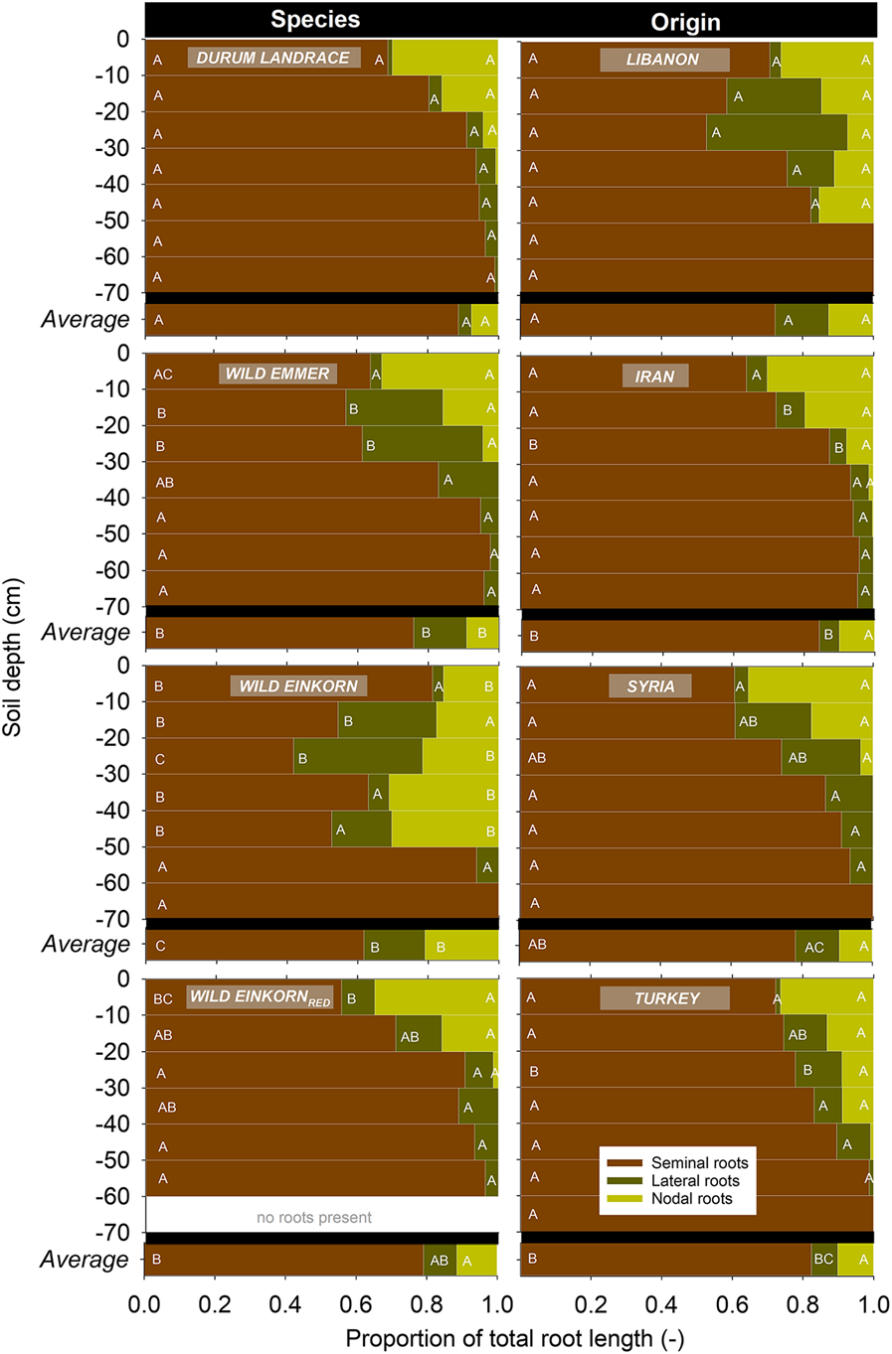
Proportion of seminal, nodal and lateral roots to the total root length. Left for the different species of the *Triticum* genus, right for different geographical origins. Different letters indicate significant differences among species at *p* < 0.005

The relative contribution of the different axes types to the whole visible root length is shown in Fig. 3. Overall, both genetic background and origin shaped the proportional length among root types at BBCH 22 ± 0.5.

Specifically, the average proportion of seminal root axes was highest in durum landraces (88.6%) and lowest in the *monococcum*-type wild einkorn (61.7%), with wild emmer (75.9%) and *urartu*-type wild einkorn_red_ (79.1%) in between. Concerning the distinction among species over depth, significant differences occurred to a depth of 50 cm, with highest differentiation in 20–30 cm (CV = 33.3%) and 40–50 cm (CV = 26.5%).

At BBCH 22 ± 0.5 lateral branches emerging from the seminal roots constituted 3.7% in durum landraces, 9.4% in *urartu*-type wild einkorn_red_, 15.0% in wild emmer and 17.4% in the *monococcum*-type wild einkorn accessions. The average higher proportion of lateral root branches in wild emmer and *monococcum*-type wild einkorn resulted mostly from stronger lateral rooting between 10 and 30 cm (durum 4.2%, *urartu*-type wild einkorn_red_ 10.5% vs. wild emmer 32.2%, monococcum wild einkorn 30.2%).

The proportion of nodal roots at early stage phenotyping was significantly different (higher) for *monococcum*-type wild einkorn (20.9%), the range for the other accessions was 9.4 ± 2.0% (not significantly different between each other). Specifically, the *monococcum*-type wild einkorn had a different depth distribution: a particularly high nodal root proportion was located from 20 to 50 cm, depth, while the other accessions had their highest nodal root proportion in the top 0–10 cm.

Concerning the role of origin, the Lebanese accessions had the lowest proportion of seminal roots (72.2%), followed by Syrian (78.2%), Turkish (82.3%) and Iranian (84.3%) accessions. The lower seminal root proportion in Lebanese and Syrian origins was due to the high contribution of lateral roots (mainly from 10 to 30 cm depth) on total root length in species collected in these countries. The proportion of nodal roots on total visible length did not show a significant effect of collection site.

#### Depth distribution of different root classes

**Fig. 4:**
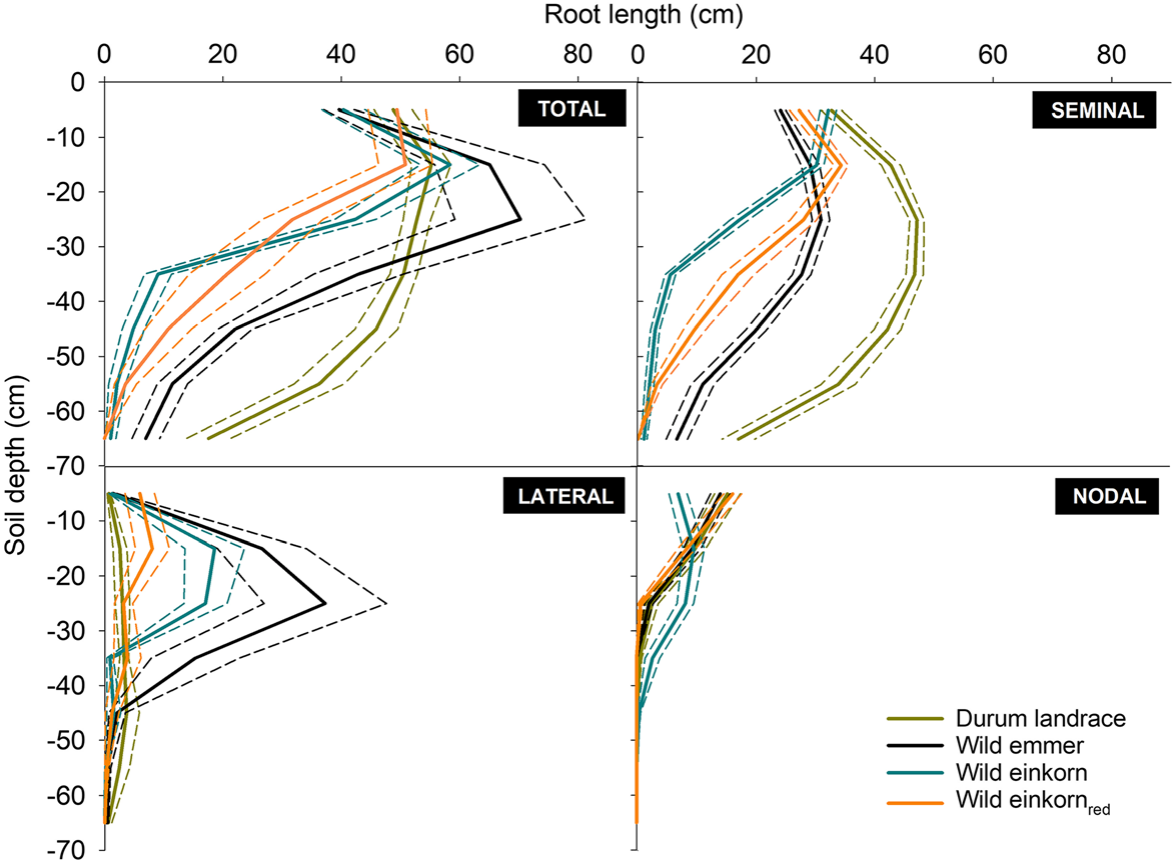
Depth distribution of visible root axes for species of the *Triticum* genus with different ploidy. **a** total root system, **b** seminal roots, **c** lateral roots, and **d** nodal roots. (Short dashed lines show the standard errors)

The depth distribution of the different visible root classes (seminal, lateral, nodal) is shown in Fig. 4.

Durum landraces and wild emmer had 53.3% (durum) and 43.6% (emmer) of seminal root lengths below 30 cm with peaks at 20–30 cm. The wild einkorn accessions (both types) on the contrary showed highest seminal root length in 10–20 cm, with 68.6% (*monococcum*-type) and 51.7% (*urartu*-type) of seminal root length in the upper 20 cm of the rhizotrons and a quick decrease towards depth.

Lateral root length of durum landraces was lowest among all species, with a rather even distribution over depth. In the upper part of the rhizotrons, it was followed by red wild einkorn (*urartu*-type), while below 40 cm this species had slightly lower lateral root length. Wild emmer showed a distinct peak in lateral root lengths between 20 and 30 cm depth, similar to the *monococcum*–type wild einkorn with a high number of lateral length between 10 and 30 cm.

Nodal roots were similarly distributed over depth for all species, except *monococcum*-type wild einkorn. While durum landraces, wild emmer and the *urartu*–type wild einkorn_red_ showed an approximately linear decrease in nodal length between 0 and 30 cm (with >50% of nodal length in 0–10 cm), *monococcum*-type wild einkorn had less nodal length in the top. *Monococcum* nodal root axes length peaked between 10 and 30 cm (63.9% of total nodal length), and then decreased to zero at 40–50 cm.

Using an asymptotic depth distribution function (high values of *β* corresponding to a greater proportion of roots with depth; Table 4), depth penetration of the different root axes types followed the order: seminal roots (average: 0.96) > lateral roots (0.90) > nodal roots (0.79). At the early phenotyping stage, differentiation in depth distribution among species was mostly driven by seminal roots with tetraploids having significantly higher *β*-values than the diploids. The trend towards higher *β*-values in the lateral and nodal roots in wild emmer and *monococcum*-type wild einkorn was not significant, still leading to an overall intermediate depth distribution of these accessions at the whole root system scale.

**Table 4:**
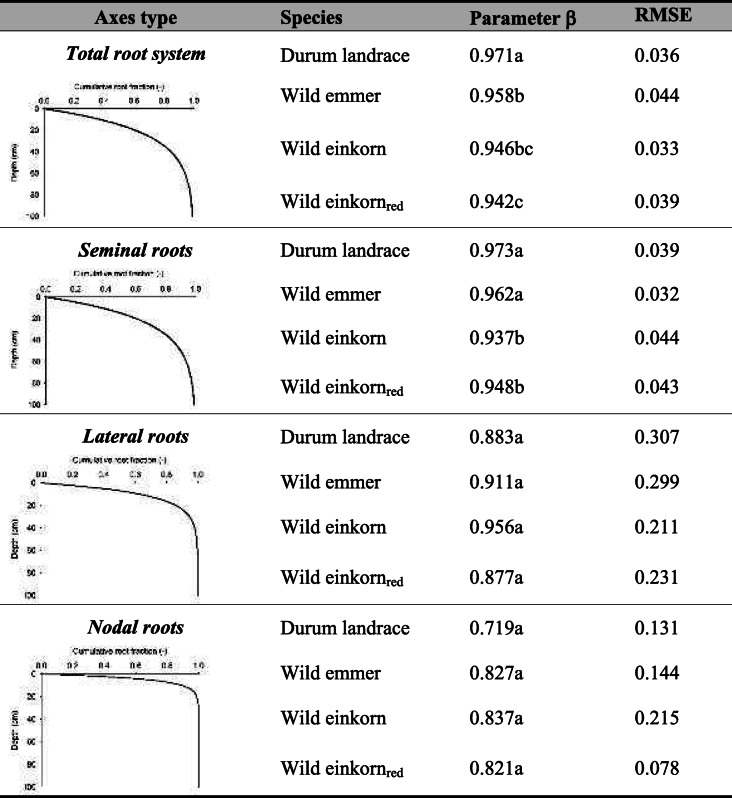
Depth distribution of root systems described by an asymptotic function of Jackson et al. (1996). The distribution parameter *β* captures the cumulative proportion of root length over depth. Model fit to the data is given by the RMSE (root mean square error; *β* parameter values with the same lower-case letter do not differ at *p* < 0.05)

The convex hull area captures the overall soil explorative capacity of the studied genotypes. It was mainly related to differences in rooting depths (R^2^ = 0.57, *p* = 0.005) which, in turn, was driven by seminal root length (R^2^ = 0.73, *p* < 0.001). Therefore, convex hull area followed a similar pattern to seminal root length: on average, the durum landraces had the highest convex area, followed by wild emmer (with a particularly high value for the Iranian accession) and both types of wild einkorn (with exception of the Syrian *uratu* accession with a comparatively large convex area). At the level of origins, Iranian accessions had highest root explorative capacity, significantly differing from Turkish and Lebanese accessions, and Syrian ones at an intermediate position.

#### Root growth of different root classes

**Fig. 5:**
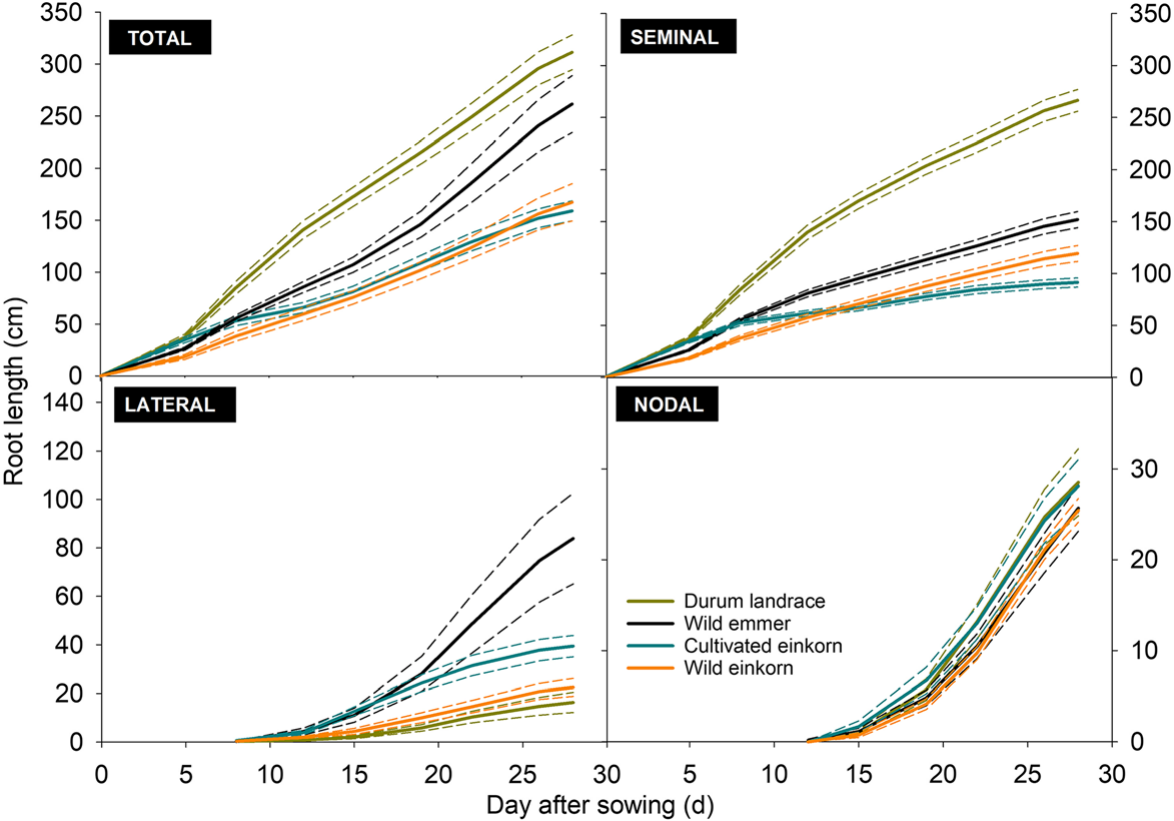
Elongation of visible root axes for species of the *Triticum* genus with different ploidy. **a** total root system, **b** seminal roots, **c** lateral roots, and **d** nodal roots. (Short dashed lines show standard errors)

Root growth (increase in visible root length) of different classes of root axes (seminal, lateral, nodal) is shown in Fig. 5.

Table 5 compares root growth dynamics between the different species based on fitted growth function parameters (modelled growth and measured values, cf. supplementary fig. S2).

**Table 5:**
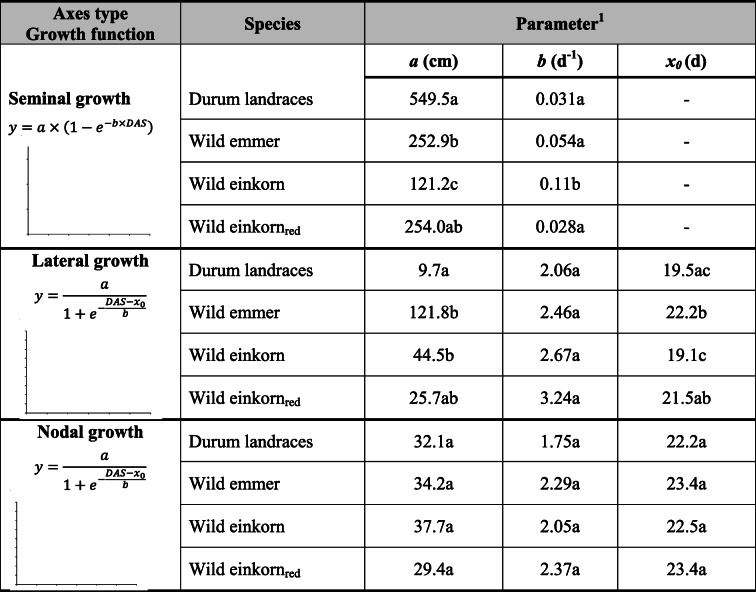
Growth functions for seminal, lateral and nodal root axes and comparison of function parameter between different *Triticum* species. (Parameter values with the same lower-case letter do not differ at *p* < 0.05)

^1^Seminal root growth is described by a function with exponential rise to maximum (used for root growth e.g. by Schnepf et al. 2018). Parameter *a* gives the maximum root length approached by the function, *b* captures the growth rate describing how the function approaches the maximum. Lateral and nodal root growth is described by a logistic growth function (as used e.g. by Gregorczyk 1991), where *a* is the maximum root length, *b* the growth rate and *x*_*0*_ is the midpoint of the sigmoid growing pattern. Curve fitting details cf. Supplementary fig. S2

Seminal growth was captured by a function with exponential rise to a maximum (average RMSE = 4.62 ± 3.52 cm; RMSE for single species cf. supplementary fig. S2). As expected from the final measured root length, the maximum values of seminal length was highest for durum landraces (differing significantly from all others, following the order durum landraces*>* wild emmer > *urartu*-type wild einkorn_red_ > *monococcum-*type wild einkorn). *Monococcum-*type wild einkorn, with lowest maximum seminal length, had the highest growth rate, significantly differing from the slower growth rate towards the maximum root length of the other species.

Lateral and nodal root growth was best captured by a three parametric sigmoidal function (RMSE_lateral_ = 0.47 ± 0.35 cm, RMSE_nodal_ = 0.25 ± 0.07 cm; for single species cf. supplementary fig. S2). Significant differences in growth parameters, however, were only found for the final lateral root length (parameter *a* in Table 4), while the dynamics in growth rate (parameter *b*) and the duration of the initial exponential stage (parameter x_0_ for day at the inflection point of the curve) did not differ significantly. Lateral roots started emerging at about eight days after sowing.

For nodal roots, all species had similar parameter values describing their growth. We notice that for durum landraces and wild emmer initiation of nodal axes was registered at 12 days after sowing, while for the wild einkorn accessions it occurred at the subsequent imaging date (15 days after sowing).

#### Root anatomy

**Fig. 6:**
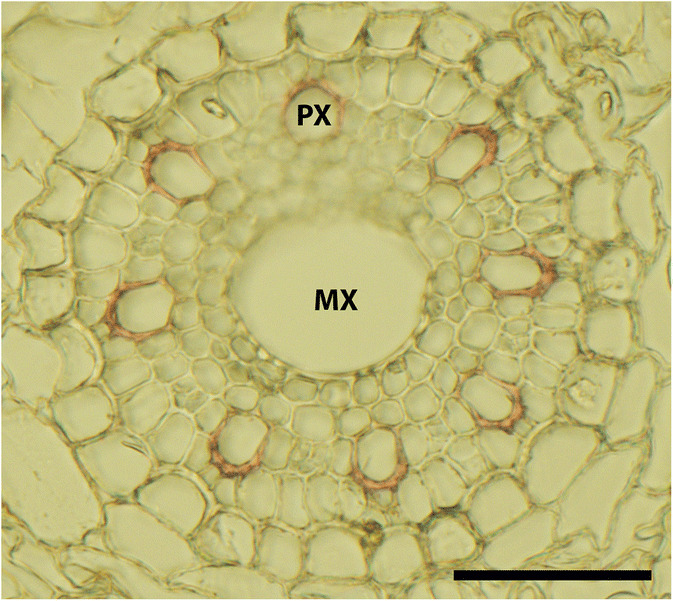
Example of a cross-section of the central cylinder (wild emmer genotype 84TK) with the central metaxylem (MX) and the peripherical protoxylem (PX) vessels. *Scale bar size: 50 mm*

Root anatomical analysis revealed significant variation in cross-sectional area of xylem vessels of seminal roots among investigated genotypes (Table 3). At the stage of measurement, anatomical samples showed both wide central metaxylem as well as small peripherical protoxylem vessels (Fig. 6). On average, there were 2 ± 1 metaxylem with a mean diameter of 45 mm and 8 ± 1 protoxylem vessels with a mean diameter of 15 mm. The average area of the protoxylem (0.0014 mm^2^) was 49.0% smaller than the metaxylem area (0.0028 mm^2^).

Differences among accessions were more pronounced in protoxylem than metaxylem. Durum wheat landraces and wild emmer had a higher number of protoxylem vessels compared to *monococcum*-type wild einkorn (*urartu* accessions were in-between). The Syrian origins had the highest number of protoxylem vessels, significantly differing from the Lebanese and Iranian origins and the Turkish having an intermediate number. Differences in metaxylem vessel number were mostly due to origin, with Turkish and Iranian accessions having significantly higher average vessel numbers compared to the Lebanese ones (Syrian in-between).

On average, the protoxylem cross-sectional area was higher for the tetraploid than the diploid species. Particularly wild emmer differed significantly from all others, while durum landraces and *urartu*-type wild einkorn_red_ had an intermediate and *monococcum*–type wild einkorn a significantly smaller protoxylem area than the other species. Origin of genotypes showed the same order for protoxylem area than vessel number (Syria>Turkey>Lebanon>Iran), with differences between Syrian vs. Lebanese and Iranian origins being significant. Metaxylem cross-sectional area did not show significant differences within the sample.

### Multivariate root system classification

**Fig. 7:**
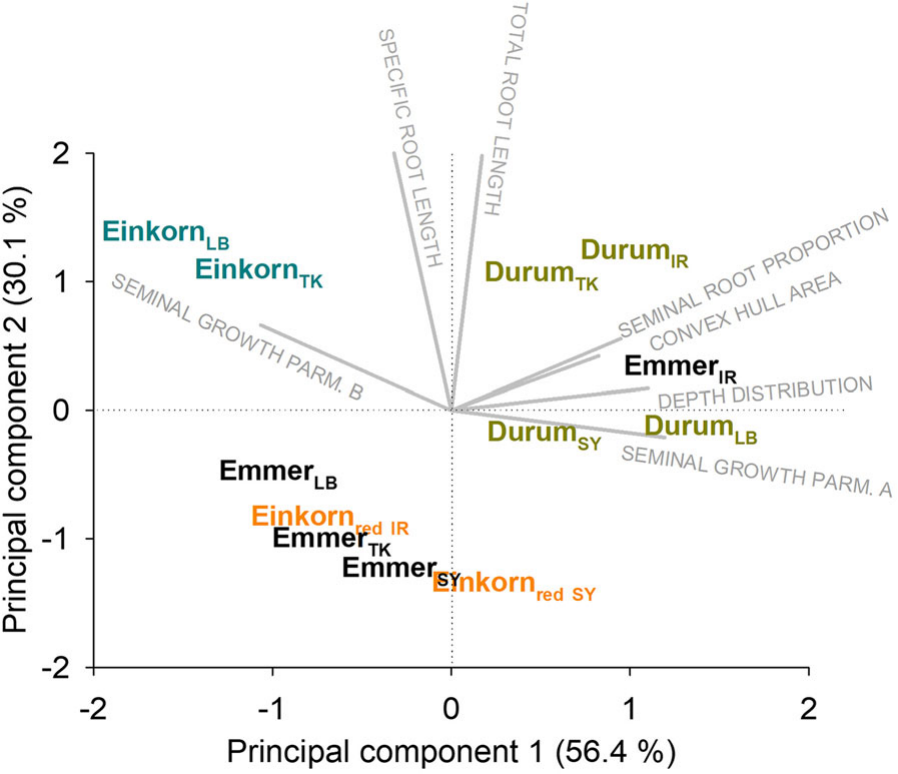
Biplots showing root trait vectors and the location of the single genotypes (species and origins; LB Lebanon, TK Turkey, SY Syria, IR Iran) according to their principal component scores

Roots are multivariate organs composed of a number of single traits that were captured/derived from phenotyping. An overall classification can be obtained by principal component (PC) analysis allocating accessions towards distinctive rooting types based in their PC scores on a biplot (Fig. 7). From the results presented above, descriptors for whole root system size (total root length, Table 3), within-root system assimilate investment (specific root length, Table 3), proportion of root axes types (seminal root proportion, Fig. 3), depth distribution (parameter *β*, Table 4), soil occupation by the root system (convex area, Table 3) and seminal root growth (parameters *a* and *b*, Table 5) were integrated in the multivariate analysis.

86.5% of the overall variability was captured by the two first principal components. PC 1 (56.4%) was mainly related to the following parameters (R values for correlation of trait with PC in parenthesis): proportion of seminal root length (81%), seminal root growth parameters *a* (98%) and *b* (83%), depth distribution parameter *β* (96%) and convex hull area (89%). PC 2 (30.1%) captured the whole root system traits of total root length (91%) and specific root length (90%).

The biplot reveals a common grouping of durum wheat landraces and the Iranian wild emmer in the (upper) right corner, sharing a positive PC 1 and a slightly negative to positive (most accessions) PC 2. This rooting type represents a large root system with intermediate SRL, strong dominance of seminal roots with deep soil penetration, thereby exploring a large soil volume. The growth rate of the dominant seminal root axes is comparatively slow, while achieving a high final length.

The two *monococcum*-type wild einkorn accessions were located in the upper left corner of the biplot with negative PC 1 and positive PC 2. Also these accessions have a comparatively large root system with a high SRL. In this rooting type, however, the lateral and nodal root axes make up a higher proportion of total root length (42.0%), leading to a shallow distribution with low convex hull area. The growth rate of the seminal root axes towards their (overall lowest) maximum length is high.

The *urartu*-type wild einkorn_red_ together with wild emmer (except the Iranian accession) formed the third rooting type, sharing a negative PC2 and a slightly positive to (for most accessions) negative PC 1. These accessions were characterized smaller root systems with low SRL. Concerning the other parameters (proportion of seminal root length, depth distribution and convex hull area, seminal root growth rate parameters), they occupied an intermediate position between the strongly seminal axes dominated rooting type (durum landraces/Iranian wild emmer) on the one hand and the root systems with higher contribution of lateral and nodal root axes (*monococcum*-type wild einkorn) on the other hand.

### Trait relations with regions of origin

Based on selected meteorological characteristics of the vegetation period at the collections sites of the accessions, correlations between traits and environmental parameters were analyzed (Table 6).

**Table 6:**
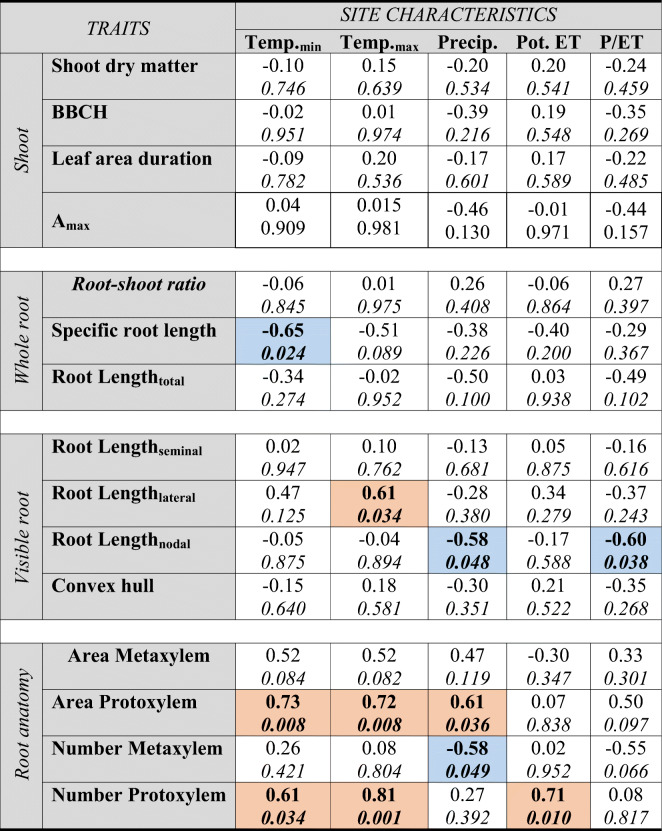
Correlation between climatic characteristics of the vegetation period at the collections sites and plant traits. (First line gives the R-values, second line [italic] the *p* value; significant correlations are highlighted in bold letters with red background color evidencing positive and blue background color negative correlations)

Temp._min_ Average of minimum temperatures, Temp._max_ Average of maximum temperatures; Precip Annual precipitation; Pot. ET Reference potential evapotranspiration (Penman Monteith); P/ET rainfall-to-potential evapotranspiration ratio

Aboveground traits did not show any significant correlation with the selected climatic variables. The strongest relation between plant traits and environmental parameters was found for the anatomical root characteristics, particularly the number and area of smaller protoxylem vessels. These traits were positively correlated with vegetation period temperature, precipitation (protoxylem area) and potential evapotranspiration (protoxylem number). The linkage between metaxylem vessel numbers with site characteristics was less evident, although a significantly negative relation was found with precipitation.

Within-root system allocation (specific root length) showed a significant negative correlation with (minimum) temperature, i.e. comparatively finer roots were associated with sites of origin characterized by lower air temperatures. Root morphological traits were also significantly correlated with climatic characteristics of the collection sites. While lateral root length was (positively) responsive to temperature, nodal root length showed a negative correlation with precipitation and the ratio of precipitation-to-potential evapotranspiration (P/ET).

## Discussion

Plant genetic resources are a important source to widen the gene pool for crop improvement, particularly aiming for better resistance against biotic and abiotic stresses (Hoisington et al. 1999). Targeted crossing requires a comprehensive trait-based characterization of potential candidates. Advances in phenotying technology have substantially facilitated the acquisition of morphological and physiological traits complementing the available genomic information for breeding (Araus and Cairns 2014).

With a focus on root system diversity, this study has investigated key distinctive traits within wheat genetic resources differing in ploidy and origin to unravel their potential contribution to breeding for resource efficient root system ideotypes (Mi et al. 2016).

### Early stage root phenotyping

Phenotyping crops beyond the seedling stage under field-near conditions is expected to improve inference towards realistic target environments (Watt et al. 2013). Beyond the phenological stage, restricted by the size of the phenotyping system, rooting patterns are also influenced by substrate properties (Wojciechowski et al. 2009). This suggest using growth media with similar hydraulic and mechanical characteristics to field soil for root phenotyping. In the context of plant root imaging, soil-based systems imply the challenge of reduced visibility (except for advanced 3D systems; Atkinson et al. 2019). However, several studies using optical (VIS, NIR) imaging of roots growing at transparent observation windows of rhizotrons have demonstrated a reliable relation between the surface-visible and total root length (Bodner et al. 2018; Nagel et al. 2012; Pfeifer et al. 2014). Also here, visible root length (mainly seminal axes) was significantly related to the whole root system extracted at the end of the experiment. We notice that there was also a significant (negative) association of specific root length (SRL) with root visibility (R^2^ = 0.56, *p* < 0.001) which was previously described by (Nagel et al. 2012). This might be related to weaker gravitropism of genotypes with a predominance of fine root axes (high SRL), possibly due to higher proportion of lateral branches with retarded gravitropic response after emergence of the higher-order roots from the main (seminal) axes (Roychoudhry et al. 2013). Within the sample, however, there was large overlap in root visibility over species and origins. Thus, our evaluations focusing at these higher scales did not show a systematic bias. Still it underlines the importance of cautious examination of possible systematic visibility effects in large and diverse phenotyping samples.

During the early phenological stages of the phenotyping experiment, the determinants of shoot dry matter were seed weight and leaf area. It is well known that seed weight is a main driver of early vigor, providing an advantage to genotypes with larger seeds via quicker emergence, leaf area expansion and superior assimilation during juvenile development (Richards and Lukacs 2002). Therefore early stage phenotyping of shoot dry matter accumulation did reflect differences in seed weight, with durum wheat landraces outperforming wild relatives due to seed-size mediated higher early vigor.

Similarly, interpretation of early stage root phenotyping has to account for phenology to interpret observations on superior root system size, particularly for monocotyledonous species. Firstly, pre-tillering root screens capturing only primary/seminal root axes can hardly predict the post-tillering root architecture with large contribution of nodal root axes to the overall size of the system (Watt et al. 2013). Secondly, during early development stages, elongation of primary/seminal roots is still influenced by seed reserves. In our experiment this was expressed by a significant relation of seminal root length with seed weight (R^2^ = 0.86; p < 0.001), which was not the case for the later emerging lateral (R^2^ = 0.05, ns) and shoot-born nodal (R^2^ = 0.08, ns.) root axes (cf. supplementary fig. S3). Thirdly, the proportion of nodal roots on the overall root length is related to tiller number (Gregory et al. 2009), which would requires continued phenotyping towards later developmental stages (post-tillering to mid shoot elongation) for proper quantitative assessment.

### Root-shoot ratio decreases from wild relatives towards durum wheat landraces

Changes in wheat breeding history have been essentially modified plant stature (height, tillering), particularly increasing harvest indices in modern compared to old genotypes (De Vita et al. 2007; Sinclair 1998). Results on the effect of dwarfing genes (Rht) on root size and partitioning of dry matter between root and shoot have been more variable. Waines and Ehdaie (2007) documented substantial reduction in root size with green revolution breeding, particularly compared to landraces. For Mediterranean conditions, Siddique et al. (1990) found more root dry-matter and higher root-shoot ratio in old compared to modern wheat genotypes. However, neither Siddique et al. (1990) nor Wojciechowski et al. (2009) identified a causal effect of dwarfing genes on root-shoot allocation patterns.

Our study revealed a major change towards higher assimilate allocation to the shoot already between wild progenitors and domesticated landraces. The higher shoot dry matter of durum landraces went along with a lower root-shoot ratio (0.12) compared to wild emmer (0.17) and wild einkorn species (0.21). Similar changes can be detected for root surface area to leaf area ratio: *monococcum-*type wild einkorn (3.81) > *urartu-*type wild einkorn_red_ (2.44) > wild emmer (2.24) > durum landraces (1.70). Decreasing trends in root-shoot ratios at the transition from diploid to tetraploid, continuing further towards hexaploid wheat species, were also reported by (Zhang et al. 2002) and (Zhao et al. 2005). It is thus likely that also shoot-to-root allometries changed upon wheat evolution, similar to modifications in allometric relations within aboveground biomass distribution of modern compared to primitive wheat species (Lv et al. 2019).

Beyond dry matter allocation at the whole plant organism level (i.e. root vs. shoot, Comas et al. 2013) pointed to the importance of considering allocation at the root organ level to be essential for drought tolerance. Species/genotypes with higher specific root length (SRL) increase soil exploration and access to water by enhanced assimilate investment into (fine) root length (McKersie and Lesheim 2013). However, the relation of SRL with drought tolerance is controversial. For example, in a sample of 23 wheat genotypes (Corneo et al. 2017) found an association between larger SRL (i.e. thicker roots) and reduce drought stress incidence as well as higher yields. This was interpreted by better soil penetration into deep layers of (seminal) root axes with higher diameter.

Among the accessions investigated here, the *monococcum*–type wild einkorn had highest SRL. The allocation pattern towards finer root axes was also positively related to the average seasonal temperature (R^2^ = 0.40). Genotypes from the most stress-prone collection site (Syria) had the most fine-root dominated root systems. Beyond the general trend towards finer root systems in plant evolution described by Ma et al. (2018), they also found a trend towards thinner roots in dryer biomes for woody species. In accordance with findings of Nakhforoosh et al. (2014) on a strong response of wheat genetic resources to water availability, also the geographical influence on SRL found in this study suggests that organ-level root plasticity is of high importance for environmental (stress) adaptation (Comas et al. 2013). Such genotypic differences in root trait expression with relevance for resource capture, e.g. root length or rooting depth, often go along with distinctive phenologies. Palta et al. (2011) for example revealed that increased early plant vigor results in larger root systems with adaptive advantages Mediterranean-type climates, while rapid exhaustion of water resources during the vegetative stage can be disadvantageous for reproductive growth where plants rely on stored soil moisture. Also in this study, similar trends in aboveground-belowground trait relations (larger seed size, enhanced early growth, larger root systems) were detected and should be considered when searching for adapted genetic resources for potential breeding target environments.

### Lower contribution of seminal root axes limits deep rooting in diploid wild relatives

There is wide consensus that deep rooting is of key importance for drought tolerance (e.g. Wasson et al. 2012; Ober et al. 2014; Becker et al. 2016), particularly in environments where crops largely rely on stored soil moisture (Tron et al. 2015). Several modelling studies have underlined the general superiority of deep rooting ideotypes in water and nitrogen (nitrate) limited environments (Postma et al. 2014). Selection of the promising accessions requires downscaling the deep rooting ideotype to the relevant underlying phenes (Lynch 2011). The importance of breeding material with traits that drive deep rooting has been recently underlined by Friedli et al. (2019) who found a decreasing trend in rooting depth over 100 years of Swiss wheat breeding (depth reached by 95% of root biomass decreased by 0.71 cm a^−1^).

Based on an asymptotic depth distribution function, our study pointed to durum landraces as most promising genetic resources for deep soil exploration compared to wild relatives. Similarly, Reynolds et al. 2006) considered (Mexican) landraces as particularly valuable genetic resources due to their deep allocation of root axes. A shallower root distribution of wild einkorn and emmer compared to modern wheat cultivars has been previously observed by Akman (2017).

For cereals, the mature root system is dominated by post-embryonic nodal root axes. However, the embryonic seminal roots (primary radicle and basal roots from coleoptilar nodes; Orman-Ligeza et al. 2013) still largely determine deep root penetration (Araki and Iijima 2001). I.e. the relative contribution of embryonic seminal vs. post-embryonic nodal roots is a driver of depth distribution of cereal root systems. In our dataset, this was evidenced by the relation between the distributional parameter *β* and the proportion of seminal root axes on total root length, which was highest in durum landraces (cf. supplementary fig. S4). Manske and Vlek (2002) identified a trend towards dominance of seminal roots and reduced tillers for modern high-yielding wheat genotypes.

For wheat genetic resources, Zhu et al. (2019) found that during wheat evolution (in a historic *T. aestivum* sample from Northwestern China) there was a tendency towards less branched and more homogeneously depth-distributed root architectures. Differences between wild vs. domesticated wheat in their length proportions of different root axes types is bound to the higher tiller numbers of wild grass species (Doust 2007). Modification of tillering pattern has therefore been used as a selection criterion in (upland) rice to shift from abundant superficial nodal roots towards deeper root penetration of seminal axes (Comas et al. 2013; Fukai and Cooper 1995).

Phenotyping in this study was constraint to a relatively early development stage with initiating nodal root growth. Although the absolute length proportions among the root axes types will evidently change with later stages (in favor of nodal root length), we still hypothesize that the early stage species differences (particularly the high nodal root length proportions of the *monococcum*-type wild einkorn; cf. Fig. 4) will be conserved/accentuated. Early appearance of nodal roots implies quick onset of assimilate competition from the growing post-embryonic tissues with the embryonic seminal root axes, leading to an overall stronger nodal root dominance (Ješko 1989; Guo and York 2019).

For lateral roots emerging from the seminal axes, the highest length (and proportion on total length) was found in wild emmer, particularly the Lebanese and Syrian accessions. The role of lateral root axes for adaptation to dry environments is controversial: Merchuk-Ovnat et al. (2017) interpreted the better performance of a near isogenic hexaploid wheat line developed from introgression of wild emmer under water stress as related to higher lateral branching. In the water efficient maize ideotype of Lynch (2013), on the contrary, lower branching frequency with higher length of the single lateral roots was postulated as advantageous. However, image analysis in this study did not allow to differentiate between lateral numbers (branching frequency) vs. single lateral elongation underlying the overall lateral length measurements.

### Wild progenitors have high initial root growth that quickly approach a maximum

Root axes in this experiment elongated quickly, with some genotypes reaching the bottom of rhizotrons (90 cm) already three weeks after sowing, which restricted the phenotyping duration to a relatively early developmental stage (BBCH 22). Root elongation rates of wheat have been reported in the range of 0.8 to 3.2 cm d^−1^, depending on the medium/bulk density (Colombi et al. 2017; Watt et al. 2003; Pritchard et al. 1987) and ambient conditions (e.g. temperature; Porter and Gawith 1999). In this experiment, the average depth penetration rate was 2.0 ± 0.5 cm d^−1^ and the average daily increase in total root length was 31.7 cm d^−1^. Comparison of these results to literature data on single axis elongation rates, however, is difficult as our observations refer to total (visible) root length. Tracking the elongation of single axes in soil filled rhizotrons is difficult as roots frequently disappear from the transparent imaging window.

Differentiation in root growth between the root axes types was higher in seminal and lateral than nodal roots (cf. Fig. 5). Growth functions were used to compare the dynamics of the measured root length time series via functional parameters. All root axes types showed a (slightly) decreasing trend in growth towards the end of the observation period, in spite of the relatively short duration of the experiment. This was expressed by the asymptotic (exponential rise to maximum) and sigmoidal patterns of seminal and lateral/nodal root growth respectively. For seminal roots, reduction in growth can be related to the onset of assimilate competition between axes types, i.e. with the initiating nodal roots (Guo and York 2019). However, the sigmoidal pattern of lateral and nodal roots also indicated the early transition to a decreasing growth rate, i.e. its midpoint x_0_ was achieved at 21 days after sowing for lateral roots and 23 days for nodal roots respectively. Here, the occurrence of artificial/mechanical growth constraints experienced by the rhizotrons grown plants cannot be excluded.

On average, the wild relatives showed higher growth rates than durum landraces (overall contrast wild vs. domesticated *p* = 0.0203). For seminal root axes this was most evident for *monococcum*-type wild einkorn. The higher early root growth vigor of wild relatives, however, went along with a low maximum length. Also for lateral and nodal axes, the wild progenitors had higher initial growth rates, although single species differences to durum landraces were not significant. It can be hypothesized that a strong root proliferation via high early root growth rates might provide an advantage to wild species establishing in a more competitive natural environment (Volis et al. 2002).

### Xylem anatomy suggests superior water transport capacity of durum landraces

Root functional properties are of high importance for improved drought resistance. This was evidenced by an early successful selection balanced root water uptake in Australian hexaploid wheat: lower seminal root xylem diameter resulted in high water availability for grain filling and higher yield in environments dependent on stored (sub)soil moisture (Richards and Passioura 1989). However, root functionality is strongly environmental context-dependent: also higher xylem conductance has been related to improved drought tolerance via more efficient root water extraction in less intense drought stress environments (Lynch et al. 2014).

There are indications that wheat evolution has been accompanied by changes in root hydraulic conductivity, increasing the water transport capacity during the transition from (wild) diploid towards (domesticated) hexaploid wheat (Zhao et al. 2005). Based on the assessment of xylem number and area, also our study provides indication of increasing water transport capacity (higher number of protoxylem vessels and xylem cross-sectional area) from diploid to tetraploid species.

The significant influence of region of origin and its climatic characteristics on root xylem characteristics (number, area of xylem vessels; cf. Table 6) underline the need for strengthening knowledge and data availability on root hydraulics in annual crops as a road towards environmental adaptation (Vadez 2014). Robust identification of causal relations between environmental site characteristics and plant (root) traits requires big datasets. This is generally difficult for root system descriptors, and in particular for root anatomy, where high throughput methods were developed only recently (Atkinson and Wells 2017). Thus our association analysis provides only an empirical indication that the environmental characteristics of sites of origin matter for understanding species differences in root characteristics.

Indirect relations of xylem diameter with root thickness might facilitate selection for an anatomical trait as reported for rice (Yambao et al. 1992). However, in our dataset these traits were only weakly correlated (with root diameter: R^2^ = 0.19 *ns*.), indicating that functionally important anatomical features remain challenging for utilization in breeding.

### Wild relatives and landraces different in root ideotype categories

Trait-based ideotypes can assist the search for genetic material for targeted crossing (Martre et al. 2015; Semenov and Stratonovitch 2013). For example, Lynch (2019) postulated two distinctive root ideotypes: (i) deep, steep and cheap and (ii) topsoil foraging. While the former optimizes (sub)soil exploration of mobile resources (water, nitrate), the latter conveys high utilization of immobile resources from topsoil (particularly phosphorus).

In our study on wheat genetic resources, integration of single root traits into a multivariate root system description via PCA revealed three rooting types: at one extreme durum wheat landraces (with Iranian wild emmer), at the other extreme *monococcum*-type wild einkorn and in-between the *urartu*-type wild einkorn_red_ and wild emmer.

**Fig. 8:**
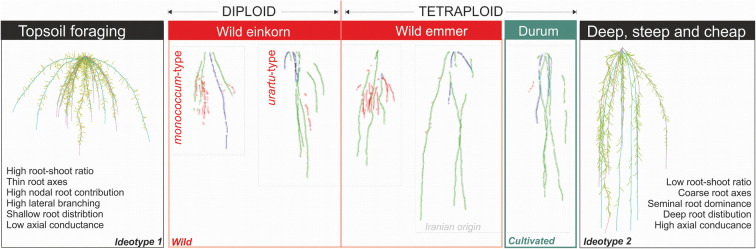
Conceptual framework for allocation of wheat genetic resources according to their root system characteristics in relation to two extreme root system ideotypes (left: topsoil foraging ideotype; right: deep, steep and cheap ideotype). Root system ideotypes have been created with the CRootBox model (Schnepf et al. 2018) varying root insertion angle, tropism strength, inter-branch distances and number of shoot-borne roots. Examples of manually tracked with GROWSCREEN-Root image analysis software (from right to left) are: Lebanese *monococcum*-type wild einkorn G3120, Syrian *urartu*-type wild einkorn_red_ SY23, Lebanese wild emmer G3100, Iranian wild emmer G1392, Syrian durum landrace B1. Light-gray frames indicate the common grouping between genotypes revealed by principal component analysis

Figure 8 provides a conceptual classification of the identified rooting types between dense and shallow growing root architectures with high topsoil exploitation at the one end and seminal root axes dominated deep growing root architectures with high subsoil exploration at the other end.

The strongly branched surface-near root systems of the *monococcum*-type wild einkorn mostly approximated the characteristics of a topsoil foraging ideotype: high dry matter allocation into fine roots, high length contribution of lateral and nodal roots, restricted deep rooting and low xylem conductance. Competitive advantages of such a rooting type has been discussed in relation to efficient exploitation of nutrient-limited (mainly P) soils (Lynch and Brown 2001; White et al. 2013).

Characteristics of a deep growing exploratory root ideotype were mostly found in the group dominated by durum wheat landraces. Their root systems had highest dominance of thicker (high SRL) seminal roots for subsoil exploration. Anatomical features (xylem area) suggested highest axial transport capacity to effectively extract subsoil water. The similarity of durum landraces from the accessions of this study with a water/nitrate efficiency ideotype agrees with the promising attributed to landraces for improving drought tolerance by (Reynolds et al. 2006).

Overall out results suggest that topsoil exploiting root systems of (diploid) wild relatives have evolved towards a subsoil exploring root systems in (tetraploid) landraces. Both constitutive and adaptive factors can be hypothesized to underlie these distinctive rooting types. The high (and quick) topsoil exploitation of diploid wild relatives could be related to:

(i) Constitutive factors of plant architecture, i.e. an intense shoot-borne nodal root system related to the abundant tillering of wild grasses, a reproductive strategy typical to wild plants under risk of grazing, herbivory or fire in natural environments (Anderson-Taylor and Marshall 1983; Yoshida and Hasegawa 1982; Doust 2007).
(ii) Adaptive factors of competition within natural vegetation communities in resource-poor soils where quick proliferation of roots into the topsoil provides a competitive advantage for acquisition of (immobile) nutrients.

The transition towards a deep soil exploring root ideotype among durum wheat landraces from dry Mediterranean environments points to:

(i) Constitutive reduction of branching (less tillering shoots; higher seminal root dominance) going along with functional/physiological changes (photosynthetic capacity of leaves, xylem transport capacity of roots; Fischer et al. 1998; Zhao et al. 2005).
(ii) Adaptation to water as main limiting resource in Mediterranean-type environments by deep root allocation for optimizing access to (sub)soil water; reduced pressure for topsoil competitive strength due to weed control and lower nutrient limitation in early agriculture.

Domestication and crop improvement has been a process of visual selection for yield and some yield-driving shoot traits (Purugganan and Fuller 2009), indirectly co-selecting changes in root and physiological features. This study has demonstrated that current phenotyping technologies can help to directly capture belowground diversity. Inserted into frameworks of root system ideotypes, these novel datasets can advance the targeted utilization of genetic resources for trait based breeding towards future cultivars with superior resource use efficiency and stress adaptation.

## Conclusion

The rooting pattern of wheat genetic resources has substantially changed at the transition from wild progenitors to cultivated landraces. Lower carbon investment into root formation relative to shoot allocation went along with structural modifications enhancing the explorative efficiency of soil resources. Wild einkorn and emmer form more superficial root architectures with higher proportion of lateral and earl-emerging nodal axes relative to the length of seminal roots. The root system of durum landraces, originating from semi-arid Mediterranean type environments, has evolved towards high soil volume exploration via deep-rooting seminal axes. *Monococcum*-type wild einkorn and durum landraces represented the two extremes, resembling the contrasting root system ideotypes of topsoil exploitation vs. deep soil exploration.

We assume that even the low-input agro-ecosystems, where landraces have evolved, substantially reduced inter-species nutrient competition compared to natural vegetation, thereby promoting deep root allocation of seminal axes which facilitated water extraction in the drought-prone regions of origin. We therefore conclude that breeding efforts for improving drought resistance using the examined accessions could mainly profit from landrace root systems. Phenotyping platforms such as Growscreen-Rhizo can facilitate screening for traits constituting the water efficient root system ideotype of landraces to better exploit root diversity for crop improvement. Root phenotyping thereby provides an empirical underpinning to further model the dynamics of observed rooting strategies for making predictions beyond the timeframe and environmental settings where observations takes place. Integration of shoot traits can further extend the relevance of root phenotyping datasets by highlighting of root-shoot allometries with relevance for field screening and trait-based breeding for specific stress environments.

## Supplementary Information

ESM 1 (DOCX 766 kb)
