# Supplementary material for: Deep soil exploration vs. topsoil exploitation: distinctive rooting strategies between wheat landraces and wild relatives
Source: Plant Soil. 2020 Dec 21;459(1):397–421. doi: 10.1007/s11104-020-04794-9 (PMC7870630; doi:10.1007/s11104-020-04794-9)
Supplement: Supplementary file 1 — (DOCX 766 kb) [file 11104_2020_4794_MOESM1_ESM.docx]

**Supplementary figures for:**

**Deep soil exploration vs. topsoil exploitation: Distinctive rooting strategies between wheat landraces and wild relatives.**

Alireza Nakhforoosh^1,2^, Kerstin A. Nagel^3^, Fabio Fiorani^3^, Gernot Bodner^1^*

^1^Division of Agronomy, Department of Crop Sciences, University of Natural Resources and Life Sciences, Vienna (BOKU), Konrad Lorenz-Straße 24, A-3430 Tulln an der Donau, Austria

^2^Global Institute of Food Security, University of Saskatchewan, SK S7N 0W9 Saskatoon, Canada

^3^IBG-2: Plant Sciences, Forschungszentrum Jülich GmbH, 52425 Jülich, Germany


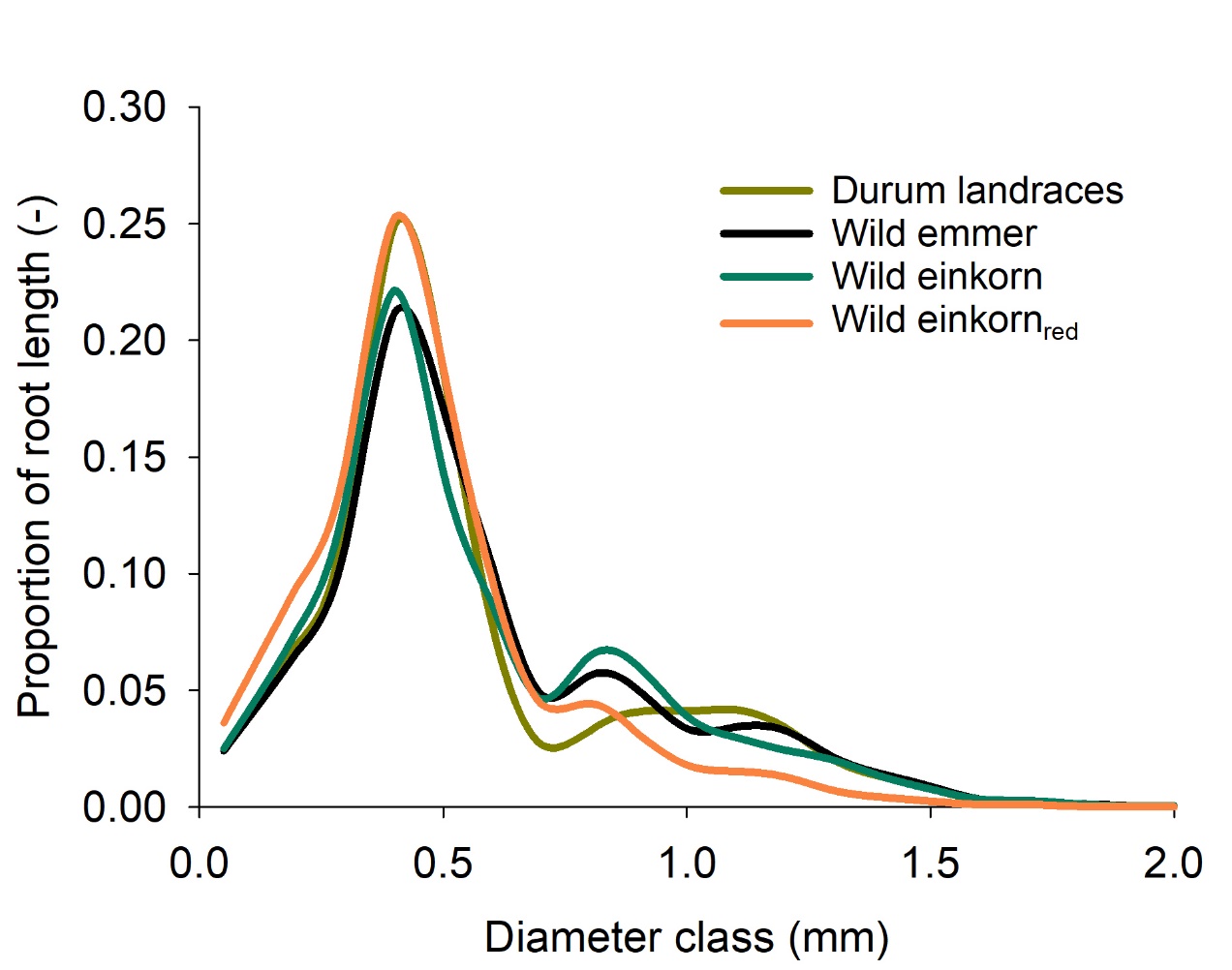


**Supplementary Figure S1.** Proportion of root length in different diameter classes measured with WinRhizo. Settings: Scanning resolution 400 dpi, diameter class width 0.2 mm.


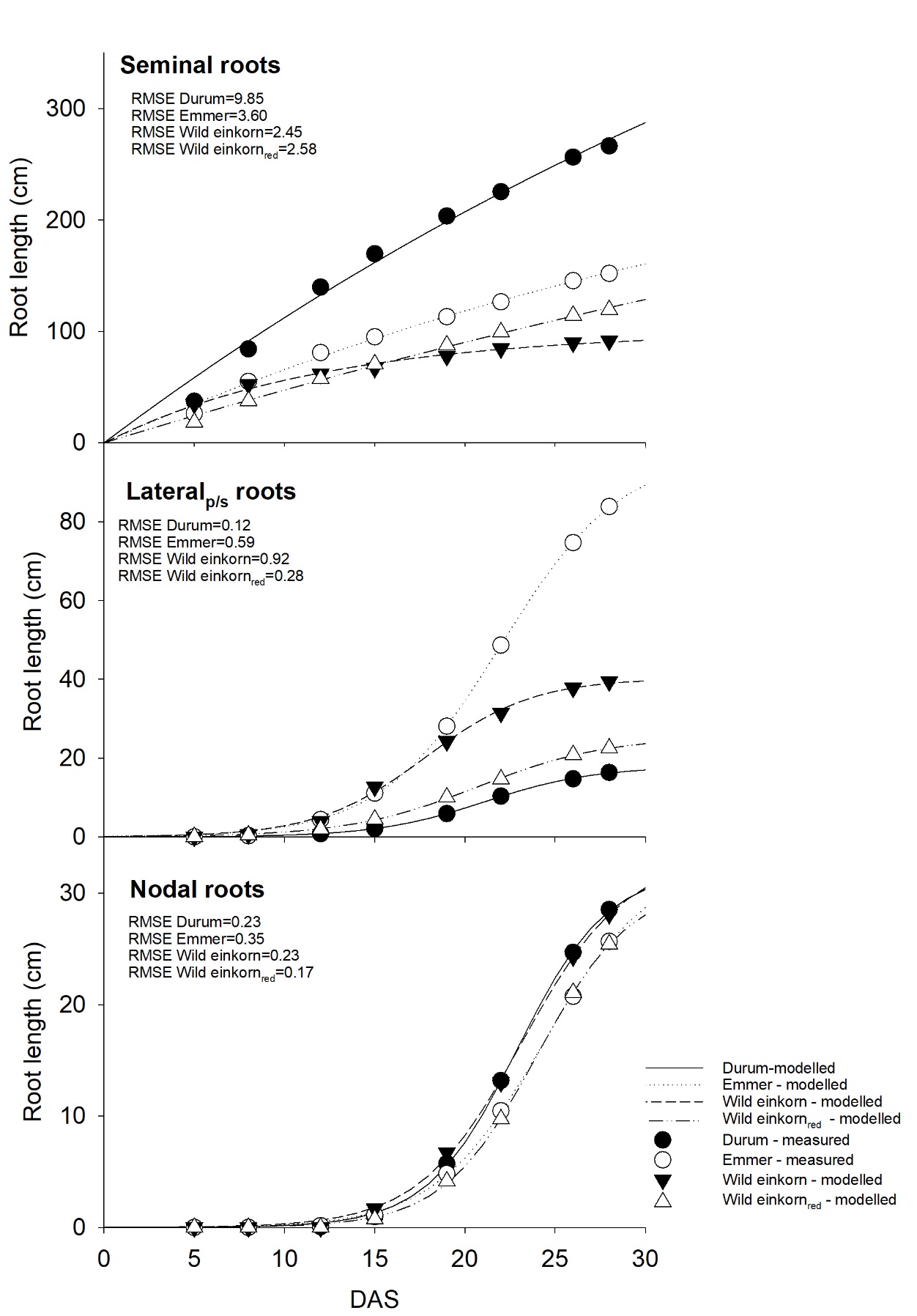


**Supplementary Figure S2.** Fitting of growth functions to measured root length of different root axes types. Symbols are measured data, lines modelled data with the fit growth functions using SAS PROC NLIN. Goodness of fit parameter RMSE is given for each species.


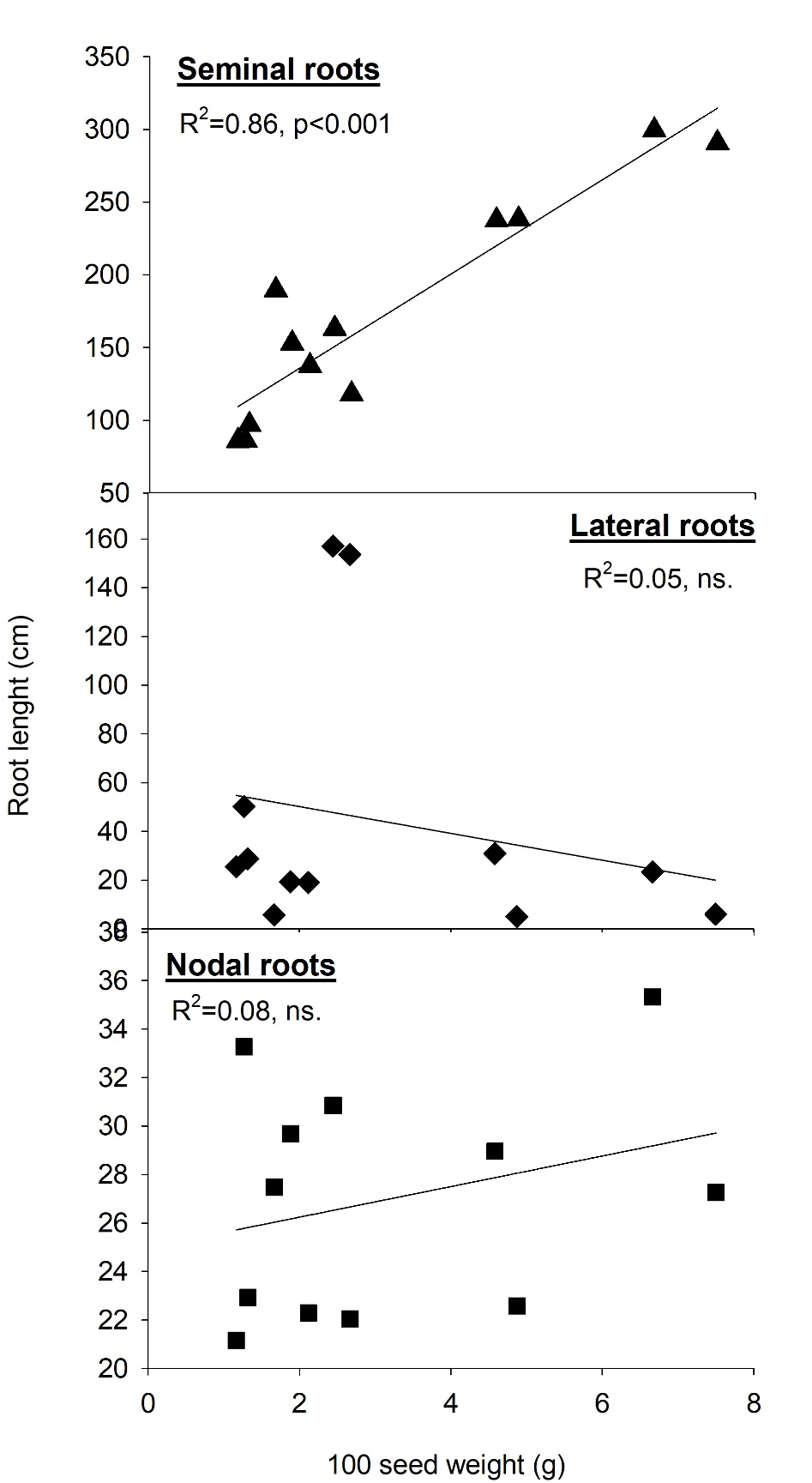


**Supplementary Figure S3.** Relation between seed weight and root length of different root axes types.


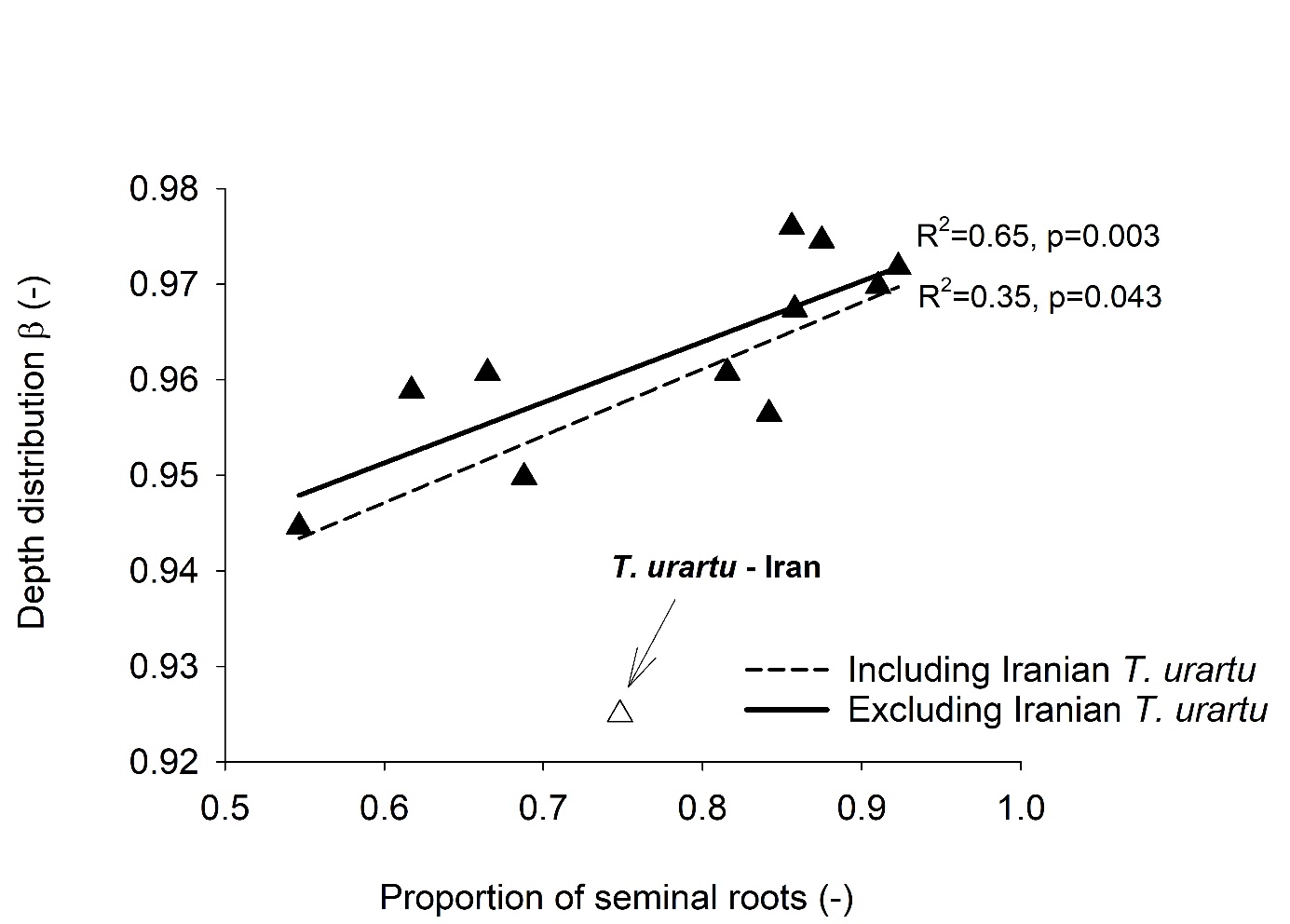


**Supplementary Figure S4.** Relation between the proportion of seminal roots on total root length and the depth distribution parameter *β* from the asymptotic root distribution model of Jackson et al. (1996).
